# Intrinsic limitations in mainstream methods of identifying network motifs in biology

**DOI:** 10.1186/s12859-020-3441-x

**Published:** 2020-04-29

**Authors:** James Fodor, Michael Brand, Rebecca J. Stones, Ashley M. Buckle

**Affiliations:** 1 Department of Biochemistry and Molecular Biology, Biomedicine Discovery Institute, Monash University, Clayton, Victoria 3800 Australia; 2 Otzma Analytics Pty Ltd, Bentleigh East, Victoria Australia; 3 Faculty of Information Technology, Monash University, Clayton, Victoria 3800 Australia; 4 School of Mathematical Sciences, Monash University, Clayton, Victoria 3800 Australia; 5 Department of Mathematics and Statistics, Dalhousie University, Halifax, Nova Scotia Canada; 6 College of Computer and Control Engineering, Nankai University, Tianjin, China

**Keywords:** Network motifs, Gene regulation, Network substructures

## Abstract

**Background:**

Network motifs are connectivity structures that occur with significantly higher frequency than chance, and are thought to play important roles in complex biological networks, for example in gene regulation, interactomes, and metabolomes. Network motifs may also become pivotal in the rational design and engineering of complex biological systems underpinning the field of synthetic biology. Distinguishing true motifs from arbitrary substructures, however, remains a challenge.

**Results:**

Here we demonstrate both theoretically and empirically that implicit assumptions present in mainstream methods for motif identification do not necessarily hold, with the ramification that motif studies using these mainstream methods are less able to effectively differentiate between spurious results and events of true statistical significance than is often presented. We show that these difficulties cannot be overcome without revising the methods of statistical analysis used to identify motifs.

**Conclusions:**

Present-day methods for the discovery of network motifs, and, indeed, even the methods for defining what they are, are critically reliant on a set of incorrect assumptions, casting a doubt on the scientific validity of motif-driven discoveries. The implications of these findings are therefore far-reaching across diverse areas of biology.

## Background

A *network motif* is a particular connected pattern of nodes and edges that appears in a network significantly more frequently than would be expected by chance. The pattern is described by the nature of the edge or lack of edge connecting each pair of nodes, making these, in mathematical terminology, “connected, induced subgraphs”. It is hypothesized that network motifs play a more important role in network function than arbitrary substructures. Since the concept was popularised in 2002 by Milo et al. [1, 2], much effort has been devoted to identifying network motifs in the hope that doing so will yield insights into network behaviour [3–5]. Network motif identification plays an important role in molecular and cell biology research, notably the study of gene regulation [3] (which describes regulatory relationships between transcription factors and their target genes), interactomes [6] (which describe protein-protein interactions), and metabolomes [7] (which describe the complete set of small molecules within a cell).

In the study of network motifs, it is critical to be able to determine whether a particular subgraph *H* (i.e. a specific connected pattern of nodes and edges) observed in a network *G* is in fact a motif, or merely a chance occurrence. In order to determine whether *H* is a motif of *G*, the following procedure (or a close variant thereof) is typically followed [8]:

- *Step 1*: Define $\mathbbm{S}(G)$ to be the set of all networks *similar* to *G*, in the sense that they have the same set of vertices, each with the same number of incoming, outgoing and bi-directional edges.
- *Step 2*: Count the number *n*_*G*_(*H*) of times that the putative motif *H* occurs in *G*.
- *Step 3*: Estimate the probability that a network selected uniformly at random from the set $\mathbbm{S}(G)$ will contain at least *n*_*G*_(*H*) copies of *H*, typically by use of an edge-switching algorithm [9]. (See the Methods section for more information about edge switching algorithms.)
- *Step 4*: If the estimated probability of at least *n*_*G*_(*H*) copies of *H* occurring by chance is less than some user-defined threshold, declare *H* to be a motif of *G*.

While the precise algorithms used to implement these steps differ between implementations, this underlying methodology is adopted by popular network motif software tools such as FANMOD [10], Mfinder [8], and MAVisto [11], and is widely used for identifying network motifs in a diverse range of biological systems. For example, Zhao et al. [12] identified 12 types of three-node motifs in chromatin-state transcriptional regulatory networks in four human cell lines. They first generated a set of 500 similar networks $\mathbbm{S}(G)$ using the FANMOD tool, and then declared a candidate to be a motif if its frequency in the original network *G* was significantly greater than in the set of similar networks, using a significance threshold (*p*-value) of 0.05. We note that these are 12 identified motifs out of only 13 motif candidates of that size in total. FANMOD was also used in identifying four types of three-node motifs in a microRNA-transcription factor (TF) co-regulatory network in non-small cell lung cancer, in this case with a candidate declared to be a motif on the basis of a 0.01 *p*-value threshold [13]. In a third example, Vinayagam et al. [14] analysed a protein-protein interaction network of *Drosophila melanogaster*, finding seven types of 3-node motifs using FANMOD to generate random sets of 1000 similar networks, with the significance of motifs evaluated using Z-scores.

These mainstream methods of motif-searching involve testing a very large number of hypotheses in parallel, with an associated high risk of false positive results. For example, the total number of possible 6-node motif candidates is over 1.5 million, meaning that if all are tested for, roughly one such candidate motif will be expected to receive a *p*-value of 10^−6^ or less purely by chance. If meaningful results are to be obtained, it is therefore critical to be able to reliably differentiate between very small *p*-values, such as between a *p*-value of 10^−6^ (likely to be a false positive in this example) and 10^−10^ (likely to be a true positive in this example). Reliably distinguishing between such small *p*-values based on empirical frequencies alone unfortunately often requires a sample size that is prohibitively large; for instance, a sample size on the order of 10 billion would be required for events with *p*-values at the 10^−10^ level to be sampled at all. As such, in order to directly evaluate the probability that a given motif candidate will occur in a network by chance, it would be necessary to generate a much larger set of similar networks from $\mathbbm{S}(G)$ than is practical using current methods. Without accurate calculation of such probabilities, however, steps 3 and 4 of the mainstream approach to motif detection cannot be performed. The customary solution to this problem has been to assume that the motif frequency *n*_*G*_(*H*) is normally distributed, thereby enabling *p*-values to be easily calculated from the corresponding Z-score, which does not require unrealistically large samples [9].

In order to explore the assumptions inherent in these mainstream methods of network motif detection, we analysed three real-world networks: the *E. coli* TF network, the *S. cerevisiae* TF network and the *E. coli* metabolic network. Both organisms are the best known and characterized genome model organisms. The mainstream methods outlined above were used in the analysis of both TF [15, 16] and metabolic networks [17, 18]. Drawing upon these empirical analyses and additional theoretical results, we investigate two assumptions that are inherent in practical implementations of this approach, namely the assumption of a normal distribution and the assumption of independence between candidates. We show that there are cases in which both of these assumptions are drastically violated, and that this has serious ramifications for present-day mainstream motif identification techniques. The final sub-section of the results discusses the problem of failing to properly distinguish motif frequency from concentration, and the implications of this distinction for motif detection methods. We conclude by arguing that these assumptions are inappropriate for the analysis of biological network motifs, as they cannot be assumed to be even approximately valid in all cases.

## Results

### The normality assumption

**Fig. 1:**
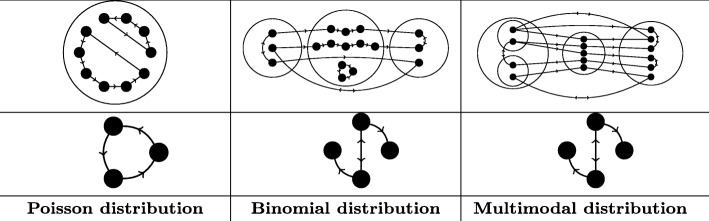
Examples of network motif distributions. Three examples of classes of synthetic networks and corresponding motifs that exhibit non-normal asymptotic frequency distributions. Top row: An illustrative network from each class. Middle row: A motif candidate. Bottom row: The form of the frequency distribution of the given motif candidate, calculated for networks drawn from the corresponding class. For full details see SI S2.2 for discussion of the Poisson distribution example, S2.3 for discussion of the binomial distribution example, and S2.4 for discussion of the multimodal example

Although motifs are commonly assumed to follow a normal distribution, as Picard et al. have observed, the normal distribution can be a poor approximation of the true motif distribution [19] (See also SI section S2.6 for other previous criticisms of motif detection methods). To illustrate this phenomenon in a wider range of cases, in Fig. 1 we explicitly identify three synthetic examples of networks and associated motif candidates that follow three completely different distributions: Poisson, binomial, and a multimodal distribution. (Detailed explanations of the example networks and proofs of the stated asymptotic distributions appear in SI sections S2.2-S2.4, and also Fig S1.) Strikingly, in this example a single subgraph follows different distributions in different networks, with the same subgraph following a binomial distribution in one class of networks (middle column) and a multimodal distribution in a different class of networks (right column). In such circumstances, assuming a normal distribution will lead to misleading *p*-values, which in this case will be highly significance-inflated. Since in these cases the underlying distribution itself is non-normal, use of improved sampling methods would not resolve these problems.

Furthermore, the usual definition of the *p*-value (the probability under the null hypothesis of obtaining results at least as extreme as what is actually observed) implicitly assumes that more extreme results are correspondingly less likely to occur. While this holds for the normal distribution, for severely multimodal distributions more extreme results can actually be *more* likely to occur than less extreme results, meaning that in such cases *p*-values calculated from Z-scores have no clear statistical interpretation (For an illustration, see Fig S2).

**Fig. 2:**
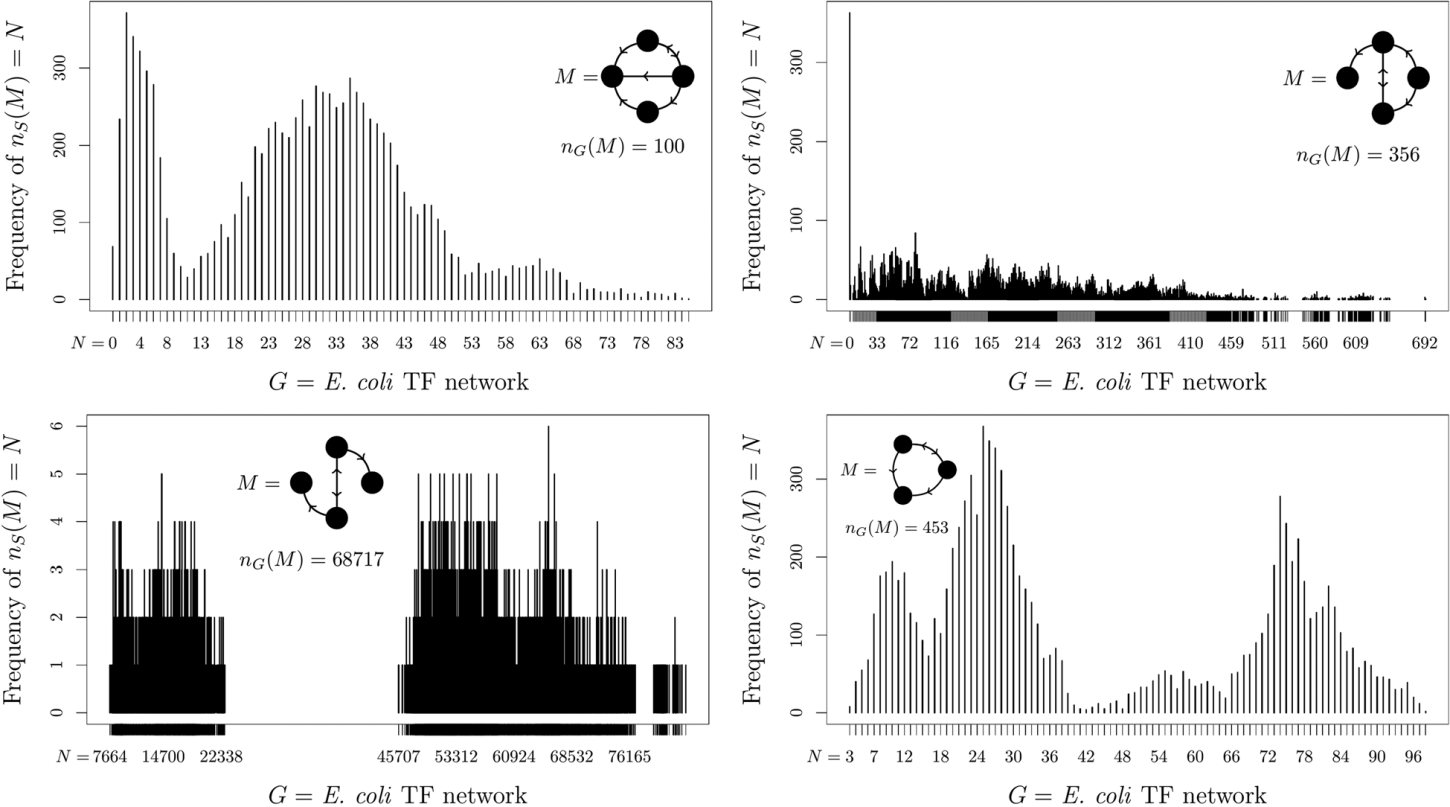
Non-normal motif-candidate frequency distributions. Example frequency distributions of the number of copies *n*_*S*_(*M*) of the candidate motif *M* found in networks similar to four bacterial transcription factor networks, as approximated by drawing 10,000 comparison networks from each $\mathbbm{S}(G)$. The actual frequency *n*_*G*_(*M*) of the candidate motif *M* in the original network is also shown for comparison

Given the variety of behaviours we observe in these simple examples, we hypothesize that real-world networks feature an unpredictable hodgepodge of behaviours governing the distribution of subgraph frequencies in a set of similar networks. Analysis of *E. coli* TF networks indeed shows this to be the case (Fig. 2; see also SI Fig S3 for more examples). While these examples all exhibit drastic departures from the normal distribution over the displayed domain, the synthetic examples from Fig. 1 complement these real-world data by showing that such non-normality can continue far into the tail of the distribution, beyond those regions which can be easily sampled.

In some cases, instead of computing *p*-values, Z-scores are used directly for significance testing [20]. For example, Kashtan et al. argued that “for large networks and subgraphs, a high cutoff of Z = 5 or 10 can be used to detect significance using the sampling algorithm. Setting the Z-score cutoff to high values is important also for avoiding false positives” [8]. While not explicitly requiring that motifs are normally distributed, this approach amounts to an implicit assumption of normality, because if the underlying distribution is not approximately normally distributed then there is no guarantee that high Z scores correspond to especially unlikely outcomes. Such misapplication of Z-score tests can give rise to grossly inaccurate results. For example, Vinayagram et al. report some extremely high Z-scores of up to 2011.9, which assuming a normal distribution corresponds to a *p*-value of approximately 10^−878959^. However, using a crude lower bound for *p* (See SI Section S2.1 for full details), any motif from the network in question must have *p* ≥ 10^−20538^. The implied *p-*value therefore falls below this lower bound by at least 858,421 orders of magnitude, indicating that for this example the implicit assumption that candidate motifs follow a normal distribution cannot possibly hold. In such cases where the normality assumption fails to hold, Z-scores cannot be used to evaluate statistical significance.

**Fig. 3:**
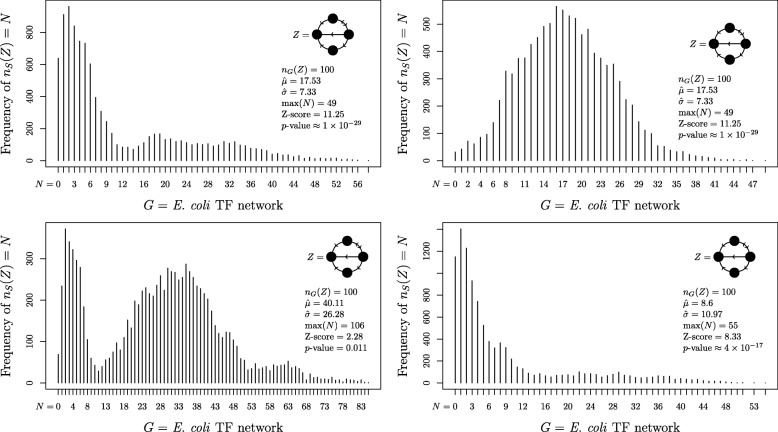
Non-reproducibility of the switching method. Frequency distributions of the number of copies *n*_*S*_(*Z*) of the motif-candidate *Z* found in networks similar to the *E. coli* TF network, as approximated by drawing 10,000 comparison networks from $\mathbbm{S}(G)$, generated using the switching method. Each of the four histograms is computed from a separate run of the algorithm, using the same candidate motif and the same input network. The actual frequency *n*_*G*_(*Z*) of the candidate motif *Z* in the original network is also shown, for comparison

In addition to not always following a normal distribution, frequency distributions of candidate motifs are not necessarily even reproducible over repeated runs. The switching method algorithm, which forms the basis for many of the most common software packages used in biological research (including FANMOD) to generate sets of similar networks, is known not to sample uniformly or independently [21], but is still commonly used because it is fast. The implicit assumption is that if sufficiently many similar networks are sampled from $\mathbbm{S}(G)$, then these drawbacks have little practical effect. We show, however, that this is not always the case, as four separate runs of the switching method on the *E. coli* TF network each produce distinctly different results (Fig. 3). Not only are the four histograms dissimilar, the estimated *p*-value for the indicated motif candidate ranges from 0.011 (not significant) to 10^−29^ (highly significant). Such widely divergent results from one run to the next render any reported *p*-values in such examples effectively impossible to interpret.

### The independence assumption

**Fig. 4:**
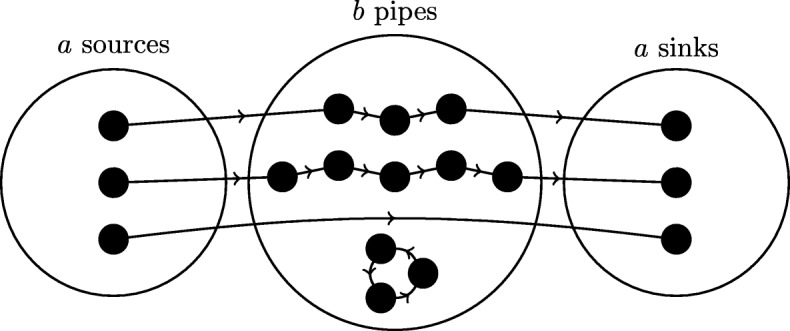
Example network showing correlated motifs. One member of a class of networks that exhibits correlation between the occurrence of different motifs

A persistent problem with multiple hypothesis testing is that hypotheses are tested individually, not jointly, with the same search procedure simply repeated for each possible motif candidate [8, 9]. Such independent testing of multiple hypotheses is only valid under the assumption that different motif candidates are distributed independently in the underlying family of similar networks $\mathbbm{S}(G)$. We show, however, that there are many circumstances in which this assumption fails to hold, and the occurrence of one motif is correlated with one or more others. To illustrate such correlation in a synthetic example, consider a network which contains three types of nodes: ‘source nodes’, with one outgoing edge and no other types of edges; ‘sink nodes’, with one incoming edge and no other types of edges; and ‘pipe nodes’, with exactly one incoming and one outgoing edge and no bidirectional edges. The class of networks considered here has *a* source nodes, *b* pipe nodes linking the source nodes to the sink nodes, and *a* sink nodes. Cyclic paths (loops) can occur among sets of pipe nodes, but not among source or sink nodes. An example of one such network is shown in Fig. 4. In this example we consider the putative motif structure *L*_3_, which is simply a 3-edge-long path.

The paths found in this network can be divided into three classes: non-cyclic (NC) paths which link source to sink via some number of pipe nodes, cyclic (C) paths which do not involve source or sink nodes, and source-sink (S) paths in which a source node connects directly to a sink without passing through any intermediate pipe nodes. We use *s* to denote the number of S-paths. Each NC-path having *l* pipe nodes contributes *l* − 1 copies of the *L*_3_ motif, while each C-path of length *l* (denoted *C*_*l*_) contributes *l* copies of the same motif as long as *l* > 4 (and zero otherwise). We also know there are *a* − *s* NC-paths: one for each of the *a* source nodes, minus one for each of the *s* S-paths. Hence, the total number of copies of the *L*_3_ motif in networks of this form is:

$$ {n}_G\left({L}_3\right)=b-a+s-3{n}_G\left({C}_3\right)-4{n}_G\left({C}_4\right) $$

where *C*_3_ is the motif that is the cycle of length 3 and *C*_4_ is the motif that is the cycle of length 4.

**Fig. 5:**
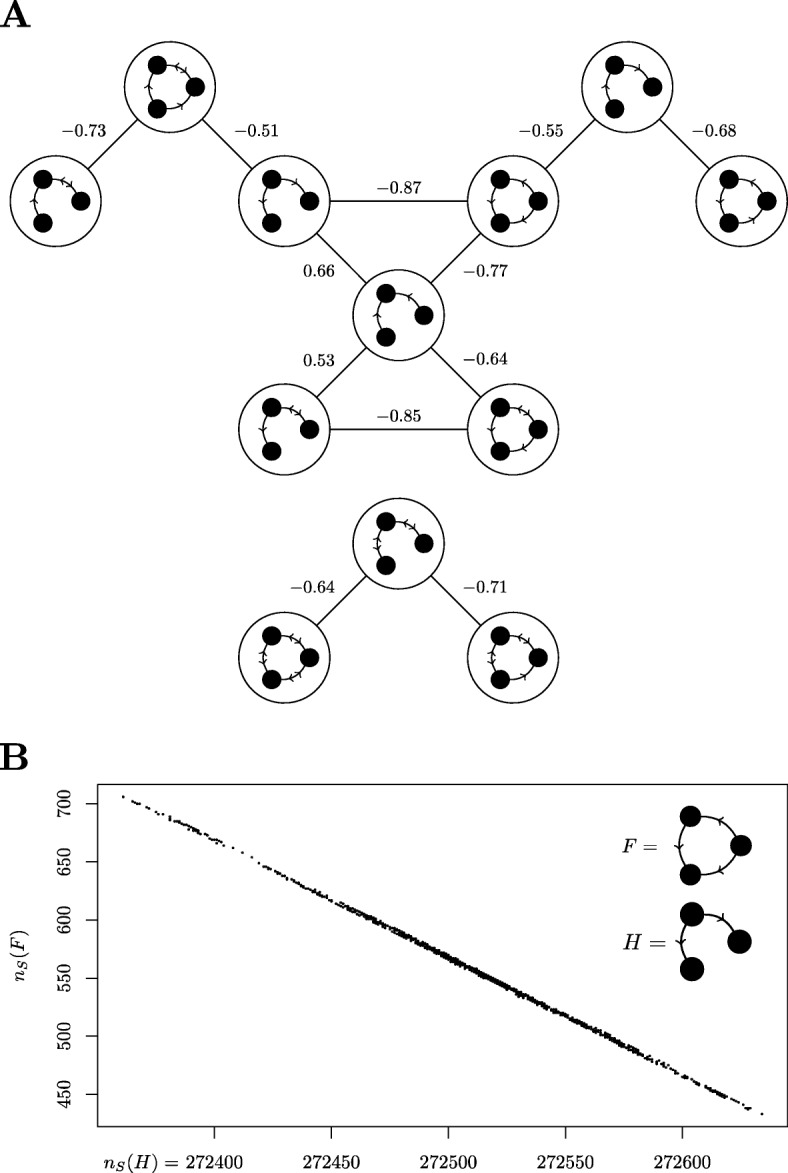
Examples of correlation between subgraphs. **a** Diagram depicting the Pearson correlation coefficients between *n*_*S*_(*H*) and *n*_*S*_(*H*^′^) for pairs of 3-node subgraphs *H* and *H*^′^. The input network is the *S. cerevisiae* TF network, and only coefficients of magnitude greater than 0.5 are shown. **b** An example of correlation in the frequency of induced subgraphs *F* and *H* in the set $\mathbbm{S}(G)$ of graphs similar to the *E. coli* TF network, with a Pearson correlation coefficient of −0.999. This is calculated by computing $\mathbbm{S}(G)$ using an algorithm based on the switching method, and then counting the number of times each of the induced subgraphs *F* and *H* appear in each graph in $\mathbbm{S}(G)$. The resulting frequencies are then plotted on the vertical and horizontal axes respectively to produce the figure shown. Note that motifs are “induced subgraphs”, i.e. they are described by the pattern created by both their edges and their non-edges. Thus, an instance of F is not a special case of H. Had motifs been defined by non-induced subgraphs, this would have created many trivial positive correlations between the frequencies of motifs and the frequencies of their own sub-motifs. Our example shows that the choice of non-induced subgraphs is not enough to avoid correlations

Given the linear relationship between *n*_*G*_(*L*_3_), *n*_*G*_(*C*_3_), and *n*_*G*_(*C*_4_), it is clear that the number of occurrences of candidate motifs *L*_3_, *C*_3_, and *C*_4_ in this type of graph is far from independent of one another. Such correlations are commonplace in real-world data, including the *S. cerevisiae* TF network (Fig. 5a) and also the *E. coli* TF network (Fig. 5b).

While it has been argued that such correlations are “artifacts of the algorithm used to generate the ensemble of randomised networks” [22], we show theoretically that this is incorrect, as correlations can occur even with uniform sampling. Whenever such correlations occur, methods which attempt to test for the existence of a wide range of different motifs without taking correlations into account will fail to deliver accurate results.

### Frequency vs. concentration

**Fig. 6:**
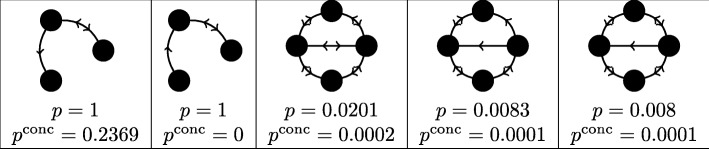
Differing frequency and concentration results. Some examples of putative motifs from the *E. coli* metabolic network where the computed frequency *p*-value *p* and computed concentration *p*-value *p*^conc^ differ considerably

Thus far we have discussed *p*-values computed on the basis of the *frequency* of occurrences of subgraphs in a network. Many available network motif detection packages, however, instead compare subgraphs based on their *concentration* in a network [9]. Concentration is calculated as the number of occurrences of subgraph *H*, *n*_*G*_(*H*), divided by the total number of connected subgraphs with the same number of nodes. In many cases this is easier to compute than the raw frequency, since concentration can be estimated by randomly sampling the network’s subgraphs, a task that does not require computing their total number. Often in the literature little distinction is made between these methods: even though the theory of network motifs largely revolves around motif frequency, concentration results are reported as though they were frequency results because this is what is most often calculated by motif-searching software. In Fig. 6, however, we present a number of cases from the *E. coli* metabolic network in which frequency and concentration statistics differ dramatically, illustrating that the choice of which method to use can drastically affect the results obtained.

**Fig. 7:**
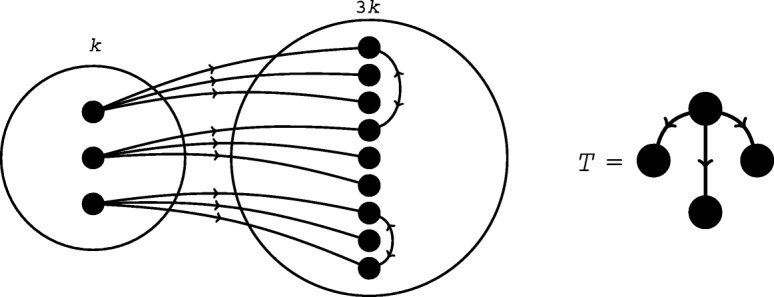
An inversion of concentration-based and frequency-based motif counting. In this network, the subgraph *T* can be a concentration-based motif only if it is a frequency-based anti-motif, and vice versa

For a synthetic example in which frequency and concentration statistics are contradictory, we consider a network *G* with *k* nodes that have exactly three outgoing and no other types of edges and 3*k* nodes that have exactly 1 incoming edge, possibly one additional bidirectional edge, and no outgoing edges. In total, the network will have *m* bidirectional edges, with *m* < 4*k*/5. One example of such a network is given in Fig. 7. We show that for any two graphs selected from the set $\mathbbm{S}(G)$, the concentration of the subgraph *T* (shown in Fig. 7) is higher if and only if the number of occurrences of *T* is lower (See SI Section S2.5 for full details). Thus, *T* can be a motif according to its *p*-value only if it is an anti-motif (meaning that it occurs *less* often than expected in the set $\mathbbm{S}(G)$) according to its *p*^conc^-value, and vice versa. Which type of *p*-value to use is therefore not merely a matter of computational convenience, but can have substantive effects on the outcome of motif analysis.

## Discussion

The ability to reliably identify network motifs is critical for understanding the complex regulatory networks that govern gene transcription and regulation, particularly in research concerning the prediction, organisation, and linking of transcription factors, binding sites, and promoters. Network motif identification can aid in overcoming the critical genomics bottleneck of processing increasingly enormous amounts of newly generated data. In addition, network motifs will play a pivotal role in the rational design and engineering of complex biological systems underpinning the field of synthetic biology. Given their importance, it is crucial that methods of identifying network motifs are statistically robust. However, we have demonstrated both theoretically and empirically that a collection of implicit assumptions present in mainstream network motif studies do not always hold, with the ramification that results from motif studies are not as mathematically unambiguous as is often presented.

All approximations used in science should be tested to determine that making these approximations does not substantively affect the results obtained. In the paper we subject typical assumptions of normally distributed motif frequencies and independence of motifs to both empirical and mathematical scrutiny, and show that they are not merely rough approximations, but grossly misleading assumptions that can lead to entirely spurious results. In particular, we showed that the assumption of normality does not always hold, and that non-normality results in materially inaccurate *p*-values, invalidating the standard methods of inferring that a given network structure is present at above-chance levels and thereby constitutes a true motif. We showed that the frequencies of different motif candidates are not always independent of one another, and that such lack of independence invalidates methods which test for the presence of large numbers of motifs independently of one another. Finally, we showed that motif frequency and motif concentration are not interchangeable, and that the choice of which to use can have substantial effects on the outcome of any analysis.

It is critical to emphasise that none of these results are simply the product of shortcomings in the sampling method being used. These faulty assumptions relate to the nature of the distribution of subgraphs, not the method by which this distribution is being sampled, and so our results hold even with perfect sampling*.* We conclude that current mainstream network motif identification methods cannot distinguish between spurious results and events of true statistical significance – a basic requirement for a mass-hypothesis testing tool.

## Future perspectives

The promise of network motifs is that sub-structures of biological relevance can be isolated purely from the structure of the network when their frequency is larger than can be explained by pure randomness, and that such a process can therefore be automated and accelerated. Underlying assumptions of this premise have been challenged multiple times in the literature (See SI S2.6.). For example, using a richer network description and following biologically-driven methods, Defoort [23] showed that structures in the network are related to the evolutionary age of the various edges, and less to their present-day topology. This echoes the results of other analyses [24–27], where it was demonstrated that the *p*-values associated with network motifs in the standard motif framework are unstable under evolutionary processes, and no evolutionary pressure for preferential selection can therefore occur on them.

While biologically-based methods are without a doubt superior to motif search that is based on frequency alone, they still do not address the need for an automatic mechanism that can be used to detect unusually frequent substructures across the burgeoning biological data. Furthermore, even given rich information for both nodes and edges, one would still hope for a statistically-defensible criterion that can reliably distinguish meaningful phenomena from statistical noise.

It is not practical within the space of a single paper to do justice to the topic of critically-analysing present mainstream network motif identification methods, and also to the separate topic of presenting viable alternatives. Nevertheless, to show that such statistically-defensible methods do exist and that improvements over existing methods are possible, in S2.7 we introduce multiple statistics regarding potential over-abundance of motifs which can all be calculated using sound statistical methods, and show that using these we are able to separate abundant motifs from non-abundant ones. These methods all relate to an idea we refer to as *anchored motifs*. An anchored motif is a motif with one vertex designated as an anchor. The anchored motif method is the method of separating the count of occurrences of each anchored motif into sub-counts that are based on the choice of vertex in the network in which the anchor is positioned. We show, with a simplistic null hypothesis and in an analysis restricted to 3-node motifs (which are by far the most frequently investigated in the literature) that the distribution of each sub-count is statistically well-understood, and its *p*-values can be calculated reliably without resorting to additional assumptions. Furthermore, the same method makes any correlations between motifs predictable, eliminating the second problem pointed to in this paper.

Additionally, the use of anchored motifs allows us to slacken the edge-degree restrictions used in standard motif methods, which are at the root of many of the unintuitive phenomena investigated in this paper. The less restrictive model makes our calculations more stable in the face of slight perturbations of the network, and so more resilient to uncertainty, to experimental error, and across evolution. It even addresses the concern by Defoort et al. [23] that the standard method limits the randomisation in generated network topologies. While the statistical behaviour of the total motif count is not as easy to model, we show multiple methods by which the various sub-counts can be combined, still yielding results that are statistically defensible.

Even though this analysis is done in a simplistic setting, we believe that the general method of anchored motifs provides a viable and statistically defensible alternative to present-day network motif detection methods. We leave it to follow-up research to present methods that combine such statistical robustness with an improved biological network model.

## Methods

### Graph definitions

Formally, a network (or graph) is a collection of nodes (or vertices) together with a collection of edges joining pairs of nodes. Typically, graphs are denoted *G* = (*V*, *E*) where *V* = *V*(*G*) is the set of nodes and *E* = *E*(*G*) is the set of edges. We consider directed graphs, where the endpoints of edges have an order, that is, an edge (*i*, *j*) ∈ *E*(*G*) is different from (*j*, *i*) ∈ *E*(*G*). If both edges (*i*, *j*) and (*j*, *i*) occur in *G*, we describe them together as a bidirectional edge. An (*i*, *i*) edge, from a node to itself, is known as a loop or a self-loop. Usually, in network motif studies, input networks are considered after all self-loops are discarded.

A subgraph of *G* = (*V*, *E*) is a graph *H* = (*V*^′^, *E*^′^) for which *V*^′^ ⊆ *V* and *E*^′^ ⊆ *E* and the edges in *E*^′^ have endpoints in *V*^′^. A subgraph is called induced if E′ = {(*i*, *j*) ∈ *E* : *i* ∈ *V*^′^and *j* ∈ *V*^′^}, that is, it includes every edge in the original graph whose endpoints are both in *V*^′^. A graph is called connected if there is a path between any two vertices (ignoring edge directions).

Note that in our analysis we exclusively consider induced subgraphs, since although some methods (such as MODA and NeMo as noted below) are capable of sampling non-induced subgraphs, most widely-used methods [28, 29], including Mfinder, FANMOD [9], and NetMODE [30] sample only induced subgraphs.

### Datasets

The following networks are included in this work.

- Transcription factor (TF) networks:
  - ◦ *E. coli* (sourced from RegulonDB [31]); 1620 non-isolated nodes, 3885 directed edges.
  - ◦ *S. cerevisiae* (yeast) TF network (sourced from Uri Alon’s website http://www.weizmann.ac.il/mcb/UriAlon/); 688 non-isolated nodes, 1079 directed edges.
- The *E. coli* metabolic network (We use the version packaged with Kavosh [32], where it is attributed to Kyoto Encyclopedia of Genes and Genomes (KEGG) [33]); 672 non-isolated nodes, 1273 directed edges.

### Network motif detection software

Many packages have been developed that enable some kind of motif detection. Mainstream packages can perform (what is sometimes called) a *k*-node subgraph census, that is, they can perform Steps 1–3 of the algorithm described in the Background section simultaneously for all *k*-node subgraphs. These packages include Mfinder [8], MAVisto [5, 11], FanMod [10, 34, 35], Kavosh [32], G-Tries [36], and NetMODE [30].

FanMod and Mfinder are packages that are widely used for biological research today, although it is also commonplace for researchers to write their own purpose-built software; see, e.g., [37, 38]. Usually input networks are assumed to be loop-free directed graphs, and a search through all (or a sample of) induced *k*-node subgraphs is performed in both the input network and the ensemble of comparison networks.

Variations of the subgraph census theme are common. For example, NeMoFinder [37] and MODA [39] only treat undirected graphs; MODA and NeMo [40] can perform a *k*-node subgraph census for non-induced subgraphs; RAGE [41, 42] focuses on 4-node subgraphs.

All experiments in this paper were performed using NetMODE, whose method for allotting Ω is as described in the Background section. In all cases, we used ∣Ω ∣  = 10,000. The reason we use NetMODE is because it has a “verbose mode” which allows studying the intermediate results (along with the final Z-scores, etc).

### Generation methods for random similar graphs

#### The configuration model

The configuration model (sometimes called the “stubs method”) is an algorithm for sampling uniformly at random from $\mathbbm{S}(G)$ that works by sampling uniformly at random from a superset *T* ⊇ *S*(*G*), then sampling again if the original sample does not belong to $\mathbbm{S}(G)$ (See, e.g., [70, Ch. 13]). The method begins with a graph *G* with *m* edges and *n* nodes, whose degree sequence is *D* = {(*c*_*i*_, *d*_*i*_)}, where *c*_*i*_ and *d*_*i*_ are respectively the out-degree and in-degree of node *i* ∈ {1, 2, …, *n*}. The aim is then to generate a new graph *G*^′^ which is sampled uniformly from $\mathbbm{S}(G)$. The algorithm achieves this as follows:

- Step 1: Allot a random permutation *β* of {1, 2, …, *m* }.
- Step 2: Create a bipartite graph *B* with *m* + *m* nodes given by the bipartition {*i*_1_, *i*_2_, …, *i*_*m*_} ∪ {*j*_1_, *j*_2_, …*j*_*m*_}, and edges determined by *β* such that for each *v* ∈ {1, 2, …, *m*}, an edge is added from *i*_*v*_ to *j*_*β*(*v*)_.
- Step 3: From *B*, generate a candidate target graph *G*^′^ by identifying the following vertices based on the input degree sequence *D*:
  - $$ {i}_1,{i}_2,\dots, {i}_{c_1} $$ and $$ {j}_1,{j}_2,\dots, {j}_{d_{1.}} $$
  - $$ {i}_{c_1+1},{i}_{c_1+2},\dots, {i}_{c_1+{c}_2} $$ and $$ {j}_{d_1+1},{j}_{d_1+2},\dots, {j}_{d_1+{d}_2} $$,
  - and so on.
- Step 4: Repeat Steps 1–3 until ${G}^{\prime}\in \mathbbm{S}(G)$
- Step 5: Return the target graph *G*^′^.

Steps 2 and 3 are illustrated in an example in Fig S9. The configuration model ensures that networks are sampled uniformly and independently, but in most real-world instances it is impractically slow.

#### The switching method

The switching method generates an ensemble, Ω, from a graph, *G*. At its core is the function described below, which generates a graph *G*^′^ from a graph *G*, such that *G*^′^ is in $\mathbbm{S}(G)$. We use the notation *x*∈_uar_*X* to indicate that *x* is sampled uniformly at random from the set *X*.

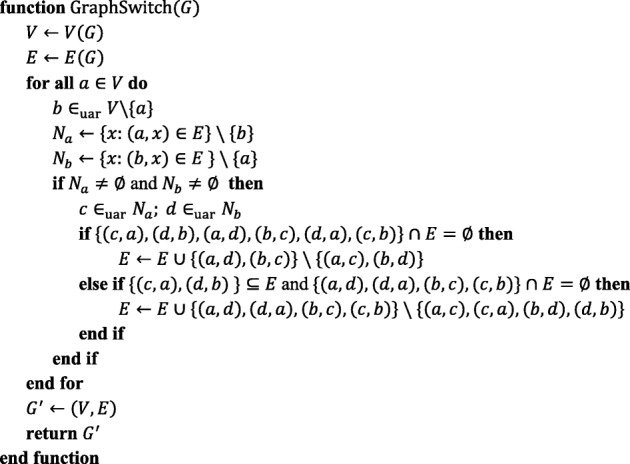



**Fig. 8:**
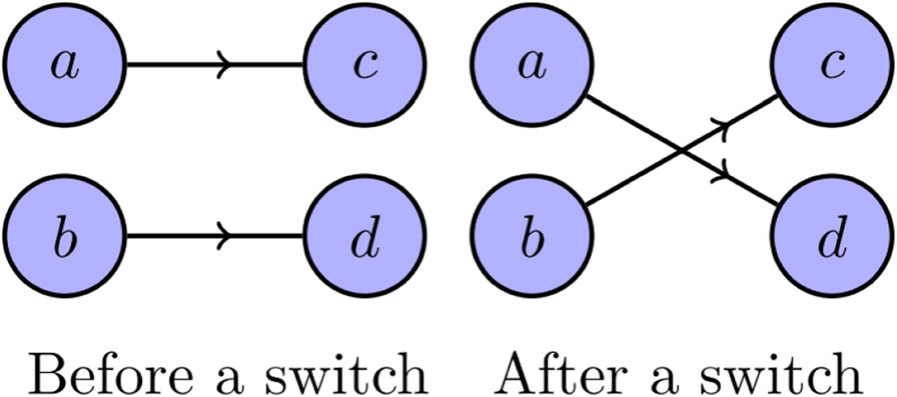
Example of a switch. The switching method, the most commonly used method for generating random graphs with a given degree sequence, utilises a Markov chain Monte Carlo (MCMC) edge switching algorithm. Given a network *G*, we choose two edges, (*a*, *c*) and (*b*, *d*), at random and switch them as depicted, provided no loops or parallel edges are generated. To sample from $\mathbbm{S}(G),$ we simply perform this operation a large number of times

A single iteration of the main loop in GraphSwitch performs the switch illustrated in Fig. 8. This is then repeated per each vertex in *G*, until creating *G*^′^. In order to create an ensemble, we take the network *G*_0_ to be *G*, the network *G*_1_ to be the output of GraphSwitch on *G*_0_, the network *G*_2_ to be the output of GraphSwitch on *G*_1_, and so on. The ensemble Ω is chosen to be {*G*_30003_, *G*_30006_, *G*_30009_, …}, with the index of the last element chosen according to the desired size of Ω. This algorithm, with the constants described here, is implemented in both Kavosh and NetMODE. The algorithm used in FanMod is similar, but instead of attempting a switch per vertex, it attempts a switch per edge.

The switching method is practical for real-world instances, but, unlike the configuration model, it does not result in a uniform sample from $\mathbbm{S}(G)$, does not guarantee independence between elements of Ω and, in fact, does not always provide a positive sampling probability for all elements in $\mathbbm{S}(G)$ (See [21] for further discussion). Figure 3 demonstrates that these drawbacks are not merely theoretical, but have wide-ranging implications on practical use of the method.

As discussed in the Results section, the outputs of the switching method are frequently not reproducible across trials. To illustrate why this is the case, consider a graph $\mathcal{G}=\left(\mathbbm{S}(G),\right\{\left(x,y\right):x\in \mathbbm{S}(G)$ and *y* is a possible output of GraphSwitch(*x*)}). This graph defines the topology over which GraphSwitch performs a random-walk. It is clear that sequentially allotted elements of Ω are highly correlated because they are close to each other in the distance metric implied by $\mathcal{G}$. A more subtle point is that the topology of $\mathcal{G}$ may be replete with small clusters that have little inter-connectedness with other clusters. When this is the case, GraphSwitch will tend to sample all of Ω from only a handful of clusters. In such a case, the high correlation between elements in Ω will not be restricted to sequential elements, but will also manifest itself in elements separated by many switches. This tendency explains the significant divergence between the histograms in Fig. 3.

Even with ∣Ω∣ = 10000, histograms of *n*_*R*_(*H*) are demonstrably non-repeatable, with the estimates of *μ* and *σ* varying widely between runs. In the figure, the *p*-value for *n*_*G*_(*Z*) is estimated once at 0.011 and once at 10^−29^. The reason for this is that although the expectation of the *p*-value is the same whether or not the samples are independent, the correlation between draws makes the effective size of Ω much smaller than 10,000, so the variance of the *p*-value estimate is unusably large.

## Supplementary information

**Additional file 1.**

## Data Availability

All data generated or analysed during this study are included in this published article and its supplementary information files.
