# Supplementary material for: Intrinsic limitations in mainstream methods of identifying network motifs in biology
Source: BMC Bioinformatics. 2020 Apr 29;21:165. doi: 10.1186/s12859-020-3441-x (PMC7191746; doi:10.1186/s12859-020-3441-x)
Supplement: Supplementary file 1 — Additional file 1. [file 12859_2020_3441_MOESM1_ESM.pdf]

Supplementary Information for "Intrinsic limitations in mainstream methods of identifying network motifs in biology"

January 11, 2020

James Fodor[a], Michael Brand[b,c], Rebecca J. Stones[c,d,e,f], Ashley M Buckle[a]

[a] Department of Biochemistry and Molecular Biology, Biomedicine Discovery Institute, Monash University, Clayton, Victoria 3800, Australia
[b] Otzma Analytics Pty Ltd, Bentleigh East, Victoria 3165, Australia
[c] Faculty of Information Technology, Monash University, Clayton, Victoria 3800, Australia
[d] School of Mathematical Sciences, Monash University, Vic 3800 Australia
[e] Department of Mathematics and Statistics, Dalhousie University, Halifax, Nova Scotia, Canada
[f] College of Computer and Control Engineering, Nankai University, China

Contents

S1 Key notation

S2 Supplementary discussion
- S2.1 Absurdly large Z-scores
- S2.2 A Poisson distribution
- S2.3 A binomial distribution
- S2.4 A multimodal distribution
- S2.5 Frequency vs. concentration
- S2.6 Previous criticisms
- S2.7 Anchored motifs

S3 Supplementary figures and legends

S1 Key notation

G	an input network (typically on n nodes).
H	a small induced subgraph; a candidate motif (typically on k nodes).
$\mathbb{S}(G)$	the set of networks similar to G.
R	a graph chosen uniformly at random from $\mathbb{S}(G)$ (denoted $R \in_{\mathrm{uar}} \mathbb{S}(G)$).
Ω	an ensemble of comparison graphs, (uniformly) sampled from $\mathbb{S}(G)$.
$f_G(H)$, $f_R(H)$	the number of copies of H in G or R, respectively.
p	probability of $f_R(H) \geq f_G(H)$.
$\hat{p}$	maximum likelihood estimation of p from Ω.
$\tilde{p}$	estimate of p from Ω based on a normal distribution assumption.
z	Z-score related to $\tilde{p}$.
$N_k(G)$	the number of connected induced k-node subgraphs in G.
$c_G(H)$, $c_R(H)$	the concentration of H in G or R, respectively.
p^{conc}	probability of $c_R(H) \geq c_G(H)$.
$\hat{p}^{\mathrm{conc}}$	maximum likelihood estimation of p^{conc} from Ω.

Table S1: The table lists some notation used frequently in the supplementary information.

S2 Supplementary discussion

S2.1 Absurdly large Z-scores

In the main text (in the section 'The normality assumption'), we give an example of a published Z-score which, assuming a normal distribution, equates to an absurd p-value. Here we describe how the corresponding p-value and the lower bound for p were calculated. This is done by use of the configuration model concept.

Theorem S2.1. *For any network G with m edges (where bidirectional edges are counted as two edges) and any induced subgraph H, if $R \in_{\mathrm{uar}} \mathbb{S}(G)$, then*

$$p := \Pr[f_R(H) \geq f_G(H)] \geq \frac{1}{m!}.$$

Proof. Since $G \in \mathbb{S}(G)$, the probability

$$p \geq \frac{1}{|\mathbb{S}(G)|}.$$

We bound $|\mathbb{S}(G)|$ by $|T|$, where $T \supseteq \mathbb{S}(G)$ is the set of graphs that can be generated at the end of Step 3 of the configuration model algorithm (See Section S4.4.1). Elements of T are determined by a permutation β of m elements, hence $|T| \leq m!$. We conclude that $p \geq 1/|\mathbb{S}(G)| \geq 1/|T| \geq 1/m!$. □

We note that the bound in Theorem S2.1 is independent of the size of H. Typically, T is much larger than $\mathbb{S}(G)$, so Theorem S2.1 only provides a crude lower bound on p.

The p-value in the main text was computed from the Z-score using the bound:

$$\begin{aligned} p &= \frac{1}{\sqrt{2\pi}} \int_z^\infty e^{-x^2/2} dx \\ &\leq \frac{1}{\sqrt{2\pi}} \int_z^\infty \frac{x}{z} e^{-x^2/2} dx \\ &= \frac{1}{z\sqrt{2\pi}} e^{-z^2/2}, \end{aligned}$$

S2.2 A Poisson distribution

We define $\mathcal{F}^t_{a,b,c}$ as the family of networks which have:

- a **source nodes**, which may have any number of outgoing edges, but no incoming edges, except possibly for one bidirectional edge.
- b **pipe nodes**, which have exactly one incoming and one outgoing edge each, and no bidirectional edges.
- c **sink nodes**, which may have any number of incoming edges, but no outgoing edges, except possibly for one bidirectional edge.
- t is the number of single-directional edges which have a source node as an endpoint.

We also introduce a family of networks, $\mathcal{I}^t_b \subseteq \mathcal{F}^t_{t,b,t}$, for which all source and sink nodes have degree 1 exactly, and there are no bidirectional edges.

In the main paper (Figure 1) we present the directed three-cycle, denoted $\vec{C}_3$, as an example of an induced subgraph whose counts follow a Poisson distribution in $\mathcal{C}_n = \mathcal{F}^0_{0,n,0}$ where n is large, as well as in a general $\mathcal{F}^t_{a,b,c}$, provided that $b \gg t^2$. In this section, we prove this claim for a general k-cycle, $\vec{C}_k$. Specifically, progressing from the special case to the general case, we prove the following claims.

Lemma S2.1. *For any $G \in \mathcal{C}_n$ with asymptotically large n and $R \in_{\text{uar}} \mathbb{S}(G)$, $f_R(\vec{C}_k)$ is Poisson-distributed with expectation $\lambda = 1/k$.*

Lemma S2.2. *For any $G \in \mathcal{I}_b^t$ with $b \gg t$ and $R \in_{\text{uar}} \mathbb{S}(G)$, $f_R(\vec{C}_k)$ is Poisson-distributed with expectation $\lambda = 1/k$.*

Theorem S2.2. *For any $G \in \mathcal{F}_{a,b,c}^t$ with $b \gg t^2$ and $R \in_{\text{uar}} \mathbb{S}(G)$, $f_R(\vec{C}_k)$ is Poisson-distributed with expectation $\lambda = 1/k$.*

Proof of Lemma S2.1. First, note that $\mathbb{S}(G) = \mathcal{C}_n$, so the choice of input network is moot. Members of $\mathcal{C}_n$ are disjoint unions of directed cycles. Each vertex, v, has an outgoing edge to exactly one vertex, u, so it is possible to consider the function defined by $f(v) = u$. This function is a permutation without fixed points or 2-cycles. In choosing $R \in_{\text{uar}} \mathbb{S}(G)$, we are effectively choosing a random permutation without fixed points or 2-cycles.

According to the theory of random permutations (See for example [1, 2]), in a random permutation on n elements, when n is large, the joint distribution function of the number of cycles of length $1, 2, \ldots, l$, for some fixed finite l, is

$$\text{Poisson}(1/1) \times \text{Poisson}(1/2) \times \cdots \times \text{Poisson}(1/l), \tag{S2.1}$$

where Poisson(λ) is the Poisson distribution with expectation λ. Importantly, Equation (S2.1) implies that the distribution of the number of k-cycles is asymptotically independent of the number of fixed points (i.e. 1-cycles) and 2-cycles. Hence the number of k-cycles in a random permutation without fixed points or 2-cycles is asymptotically distributed Poisson($1/k$). □

In particular, this result implies that for an asymptotically large n

$$\begin{aligned} |\mathcal{C}_n| &\approx n! \times \Pr[X_1 = 0] \times \Pr[X_2 = 0]\,; X_1 \sim \text{Poisson}[\lambda = 1]; X_2 \sim \text{Poisson}[\lambda = 1/2] \\ &= n! \times e^{-1} \times e^{-0.5} = n! e^{-1.5} \end{aligned} \tag{S2.2}$$

Proof of Lemma S2.2. Let $G \in \mathcal{I}_b^t$, and label the sources $S_1, S_2, \ldots, S_t$ and the sinks $F_1, F_1, \ldots, F_t$. An example is given in Figure S4.

Notably, in this network family each of the b pipe nodes can either be part of a cycle composed entirely of pipe nodes or part of a path beginning with some S_i and ending in some F_j. Note also that relabelling the sink nodes, so that each path beginning with S_i ends with the corresponding F_i makes no change to the validity of the network or its cycle structure. There are always exactly $t!$ networks that map in this way into any specific network, this being the number of possible permutations in connecting S nodes to F nodes, so we can assume, in this special case, that all paths are S_i-to-F_i paths, without this changing the probability to have any specific number of cycles.

The probability of having exactly s copies of $\vec{C}_k$ in $R \in_{\text{uar}} \mathbb{S}(G)$ is

$$\sum_{n=0}^{b} \Pr[\text{exactly } n \text{ nodes in } R \in_{\text{uar}} \mathbb{S}(G) \text{ are in cycles}] \times \Pr\Big[f_R(\vec{C}_k) = s; R \in_{\text{uar}} \mathcal{C}_n\Big]$$

In Lemma S2.1, we showed that for $R \in_{\text{uar}} \mathcal{C}_n$ the probability of $f_R(\vec{C}_k) = s$ is Poisson with parameter $\lambda = 1/k$ and independent of n for a sufficiently large n. In other words, for every desired level of accuracy, there is some N, such that for $n \geq N$ the Poisson approximation satisfies the accuracy requirements. We claim that in taking $b/t \to \infty$, the probability that the number of nodes in cycles is at least N, for any constant N, becomes arbitrarily close to 1, so in the case of networks in $\mathbb{S}(G)$ the Poisson distribution holds as an asymptotic bound, just as it does for networks in $\mathcal{C}_n$.

To see that the probability of $n \geq N$ approaches 1, consider the number of distinct similar networks in $\mathbb{S}(G)$ that have exactly n pipe nodes in cycles. This is

$$\binom{b}{n}|\mathcal{C}_n|\frac{(b-n+t-1)!}{(t-1)!}, \tag{S2.3}$$

where the expression $(b-n+t-1)!/(t-1)!$ is the number of ways in which $b-n$ named elements can be placed in t named bins. By utilising the approximation $|\mathcal{C}_n| \approx n!/e^{1.5}$ (from Equation (S2.2)), the expression becomes

$$e^{-1.5}b!\binom{b-n+t-1}{t-1}.$$

Summing this value over $n \geq N$, the result is[1]

$$e^{-1.5}b!\binom{b-N+t}{t},$$

of which the special case $N = 0$, approximating $|\mathbb{S}(G)|$, is

$$e^{-1.5}b!\binom{b+t}{t}. \tag{S2.4}$$

We conclude that

$$\Pr[n \geq N] \approx \frac{\binom{b-N+t}{t}}{\binom{b+t}{t}} \geq \left(\frac{b-N}{b+t}\right)^t. \tag{S2.5}$$

Keeping N as a constant and taking $b/t \to \infty$, the probability approaches 1, and the Poisson asymptotic distribution is reached, as desired. □

In generalising from $\mathcal{I}_b^t$ to all of $\mathcal{F}_{a,b,c}^t$, we will also be interested in the special case where we condition on all t paths being non-empty.

Whereas the number of ways in which x named elements can be placed in y named bins is $(x+y-1)!/(y-1)!$, the number of ways in which this can be done conditioned on all y bins being nonempty is only $x!\binom{x-1}{y-1}$. Introducing this change in Equation (S2.3) leads to

$$\binom{b}{n}|\mathcal{C}_n|\frac{(b-n-1)!}{(t-1)!}.$$

Following it through to Equation (S2.5) then leads to

$$\Pr[n \geq N] \approx \frac{\binom{b-N}{t}}{\binom{b}{t}} \geq \left(\frac{b-N-t}{b}\right)^t, \tag{S2.6}$$

which also approaches 1 as $b/t \to \infty$. Hence, the Poisson distribution is the asymptotic distribution of cycle frequency also in this case.

Proof of Theorem S2.2. So far, our calculations have been applicable exclusively to $\mathcal{I}_b^t$. In generalising to $\mathcal{F}_{a,b,c}^t$, we are interested in the asymptotic case where b/t^2 is taken to infinity (regardless of a and c).

Consider what a network $G \in \mathcal{F}_{a,b,c}^t$ looks like, and, in particular, how the pipe nodes are arranged. These can be either in cycles or in one of t paths connecting the source nodes to the sink nodes. Some of these t paths may be empty, in the sense that a source node is connected directly to a sink node, with no pipe nodes in between. This concept is illustrated in Figure S5.

[1] We use here $\sum_{n=N}^{b}\binom{b-n+t-1}{t-1} = \binom{b-N+t}{t}$. This equation, or, more generally, $\sum_{i=0}^{x}\binom{i+y}{y} = \binom{x+y+1}{y+1}$, is a trivial and well-known corollary of the easy-to-verify equality $\binom{y}{x} + \binom{y}{x+1} = \binom{y+1}{x+1}$.

Looking only at the pipe-node arrangement, a general $G \in \mathcal{F}^t_{a,b,c}$ looks very much like the special case $\mathcal{I}^t_b$. The main difference is that the t paths of pipe nodes are no longer labelled by an index i. Rather, they are interchangeable.

We will, therefore, designate an order on these paths. For example, we can sort the paths lexicographically by the identities of the pipe nodes composing them.

To specify the network completely, we must list for each source and each sink node which other nodes they connect to. However, we can do this simply by listing for each source and each sink node which of the t paths they connect to. This can be described in the form of two partitionings of the t paths, one specifying which paths connect to each source node, the other describing which paths connect to each sink node. Additionally, we must specify the arrangement of bidirectional edges between source nodes and sink nodes.

In any case where the pipe nodes are organised in such a way that all t paths are nonempty, any pair of partitionings of the t paths to subsets with appropriate cardinalities, combined with any valid arrangement of the bidirectional edges, yields a valid network. As such, if we condition on all t paths being nonempty, the arrangement of the pipe nodes is independent of the arrangement of the sources and sinks. (Even though the sources and sinks may be mutually dependent, due to bidirectional edges.) As such, the scenario is equivalent to the one described in Lemma S2.2, leading to Equation (S2.6) and a Poisson distribution.

If some of the paths are empty, on the other hand, this may lead to certain partitioning pairs not yielding a valid network (e.g. by connecting the same source to the same sink through multiple empty paths) or may lead to a situation in which two distinct pairs of partitionings lead to the same network configuration. (See Figure S6 for an example of how this can occur.)

Both issues described cause particular configurations of the pipe nodes that include empty paths to receive a lower probability than configurations that have no empty paths. As a result, the probability that none of the paths are empty is certainly no larger than it is under the assumption of independence between pipe nodes and other nodes. We can therefore calculate the probability that none of the paths are empty in $\mathcal{I}^t_b$, and use it as the lower bound for the probability in any $G \in \mathcal{F}^t_{a,b,c}$.

The total number of pipe-node configurations in $\mathcal{I}^t_b$ is given in Expression (S2.4). Upon conditioning on all t paths being nonempty, the remaining number is only $e^{-1.5}b!\binom{b}{t}$. The probability that all t paths are nonempty is therefore bounded from below by

$$\frac{e^{-1.5}b!\binom{b}{t}}{e^{-1.5}b!\binom{b+t}{t}} = \frac{b!^2}{(b-t)!(b+t)!} \geq \left(\frac{b-t}{b}\right)^t \geq 1 - \frac{t^2}{b}. \tag{S2.7}$$

Taking $b/t^2 \to \infty$, this probability becomes arbitrarily close to 1. Hence, the asymptotic distribution in the general case is Poisson, as when conditioning on all paths being nonempty. $\square$

S2.3 A binomial distribution

Let $\mathcal{B}^t_b \subseteq F^t_{t,b,t}$ be the family of graphs where each source has exactly one outgoing edge and exactly one bidirectional edge, whereas each sink has exactly one incoming edge and exactly one bidirectional edge. In the main paper (Figure 1), we claim regarding the induced subgraph $M =$ 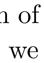 that the distribution of its count in $\mathcal{B}^t_b$ is asymptotically binomial. In this section we prove this claim. Technically, the claim we prove is as follows.

Theorem S2.3. *When $t \to \infty$ and $\frac{b}{t^2} \to \infty$, the distribution of the number of appearances of the induced subgraph $M =$ 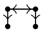 in $R \in_{\text{uar}} \mathbb{S}(G)$ for $G \in \mathcal{B}^t_b$ tends to a binomial distribution with expectation $t/4$ and standard deviation $\sqrt{t}/4$. Formally:*

$$\lim_{t\to\infty} \lim_{\beta\to\infty} Pr\left[\frac{f_R(M) - t/4}{\sqrt{t}/4} \leq t : R \in_{\text{uar}} \mathcal{B}^t_{\lceil \beta t^2 \rceil}\right] = \phi(t) \tag{S2.8}$$

where ϕ is the cumulative distribution function of the standard normal distribution.

Proof. Preliminaries: Once again, we note that $\mathbb{S}(G) = \mathcal{B}_b^t$, so the choice of input network is moot.

Let $b = \lceil \beta t^2 \rceil$. As shown in Equation (S2.7), by taking $b \gg t^2$ we ensure that the probability that all t pipe paths are nonempty approaches 1, making the distribution of bidirectional edges independent of the distribution of unidirectional edges. This being the case, we can ignore the unidirectional edges and their configuration in our analysis, noting that the number of appearances of M is simply the number of bidirectional edges from a source to a source. From this observation, the number of distinct bidirectional edge arrangements in graphs in $\mathcal{B}_b^t$ that have exactly k copies of the M induced subgraph is

$$\left(\binom{t}{2k}\frac{(2k)!}{k!2^k}\right)^2 (t-2k)! = t!^2 \frac{1}{(t-2k)!k!^2}\frac{1}{4^k}. \tag{S2.9}$$

Explanation: We need to pick out $2k$ of the t source nodes, and pair them up using bidirectional edges. There are $\binom{t}{2k}$ ways to select them and $\frac{(2k)!}{k!2^k}$ ways to pair them. We then have $t-2k$ source nodes left, each of which needs to be connected to a sink node. We therefore need to pick $t-2k$ sink nodes, and decide which source to pair up with which sink. There are $\binom{t}{2k}$ ways to pick the sink nodes, and $(t-2k)!$ ways to match sources with sinks. This leaves us with $2k$ sink nodes that need to be paired up, this having $\frac{(2k)!}{k!2^k}$ possibilities.

Let us fix t to a value satisfying $t > 3$ and $t \equiv 3 \pmod 4$. For a constant t, Equation (S2.9) is proportional to

$$P(k) \stackrel{\text{def}}{=} \frac{t!}{(t-2k)!k!^2}\frac{\sqrt{\pi}}{4^k}\frac{\sqrt{t-1}}{2^t}.$$

Let us define $B(n,i) \stackrel{\text{def}}{=} \binom{n}{i}\frac{1}{2^n}$, which is the probability of a random variable $X \sim \text{Binom}[n; p = 1/2]$ to have the value i. We will prove

$$k \leq \frac{t-1}{4} \Rightarrow B(t,2k) \leq P(k)/2 \leq B(t+1, 2k+1), \tag{S2.10}$$

$$k \geq \frac{t-1}{4} \Rightarrow B(t,2k) \geq P(k)/2 \geq B(t+1, 2k+1). \tag{S2.11}$$

For $k = 0$, this can be checked directly. For all other values, we prove the claim by use of Stirling's Formula in its bounded form:

$$n! = \sqrt{2\pi n}\left(\frac{n}{e}\right)^n e^{\lambda_n} \text{ with } \frac{1}{12n+1} < \lambda_n < \frac{1}{12n}.$$

We begin by proving $k \leq (t-1)/4 \Rightarrow B(t,2k) \leq P(k)/2$. Substituting Stirling's Formula in, to estimate $(2k)!$ and $k!$, we reach that

$$\frac{P(k)/2}{B(t,2k)} = \binom{2k}{k}\frac{\sqrt{\pi}}{4^k}\frac{\sqrt{t-1}}{2} = \frac{\sqrt{t-1}}{2\sqrt{k}}e^{\lambda_{2k}-2\lambda_k}.$$

We assumed $k \leq (t-1)/4$, but note that due to our choice of t, $k \leq (t-1)/4$ implies $k \leq (t-3)/4$. If $k \leq (t-3)/4$, this ratio is bounded from below by

$$\sqrt{\frac{4k+2}{4k}}e^{\lambda_{2k}-2\lambda_k} \geq \sqrt{1+\frac{1}{2k}}e^{\frac{1}{24k+1}-\frac{1}{6k}}.$$

It is not difficult to ascertain that this value is greater than 1 for all natural k.

Similar logic can be followed to prove all four conditions in Equations (S2.10) and (S2.11).

Having established Equations (S2.10) and (S2.11), we know that

$$\sum_{k\leq\frac{t-1}{4}} B(t,2k) + \sum_{k>\frac{t-1}{4}} B(t+1,2k+1) \leq \sum_k P(k)/2 \leq \sum_{k\leq\frac{t-1}{4}} B(t+1,2k+1) + \sum_{k>\frac{t-1}{4}} B(t,2k).$$

The binomial distribution has the normal distribution as a known asymptotic limit, so when $t \to \infty$, these inequalities become

$$\frac{1}{2}\,\phi\left(\frac{\left(\frac{t-1}{4}\right)-\left(\frac{t}{2}\right)}{\left(\frac{\sqrt{t}}{2}\right)}\right)+\frac{1}{2}\left(1-\phi\left(\frac{\left(\frac{t-1}{4}\right)-\left(\frac{t+1}{2}\right)}{\left(\frac{\sqrt{t+1}}{2}\right)}\right)\right)\leq\sum_k P(k)/2,$$

and

$$\sum_k P(k)/2 \leq \frac{1}{2}\,\phi\left(\frac{\left(\frac{t-1}{4}\right)-\left(\frac{t+1}{2}\right)}{\left(\frac{\sqrt{t+1}}{2}\right)}\right)+\frac{1}{2}\left(1-\phi\left(\frac{\left(\frac{t-1}{4}\right)-\left(\frac{t}{2}\right)}{\left(\frac{\sqrt{t}}{2}\right)}\right)\right),$$

where the reason we use $\frac{1}{2}\phi(\cdots)$ instead of $\phi(\cdots)$ is that we either only pick even values or only pick odd values from the binomial distribution, and therefore we reduce the total probability by a factor of 2.

Applying the Sandwich Theorem, we reach $\sum_k P(k)/2 = 1/2$, meaning that $P(k)$ sums to 1 and is therefore a proper distribution function.

The binomial distribution is known to converge to a normal distribution in the sense of Equation (S2.8), so by applying the Sandwich Theorem on Equations (S2.10) and (S2.11), we conclude the correctness of the theorem subject to $t \equiv 3 \pmod 4$, and by monotonicity also without this restriction. □

S2.4 A multimodal distribution

In the main paper (Figure 1), we claim regarding the induced subgraph $M =$ 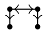 that its frequency distribution in $\mathbb{S}(G)$ is multimodal for $G \in \mathcal{G}^r_{a,b}$, where $\mathcal{G}^r_{a,b}$ is defined as the subset of $F^t_{a,b,t}$ where (excluding bidirectional edges), precisely $2r-2$ sources have out-degree 1, and the other sources have out-degree $r > 1$, each sink is the endpoint of exactly one incoming edge, and each source and each sink is the endpoint of exactly one bidirectional edge. We show in this section that the frequency distribution includes a large and asymptotically unbounded number of modes (a phenomenon we refer to as "extreme multimodality") and, furthermore, the probability for each mode may be many orders of magnitude more than the probability for the troughs between modes.

Some empirical data for real-world networks appear to have these properties; see Figure S3. However, it is impossible, given empirical data alone, to make the claim that this behaviour runs through the entire tail of the distribution. In this section, we prove the multimodality properties of the count distribution of M analytically.

We consider the family $\mathcal{G}^r_{a,b}$ (as defined above), denoting t as the number of sinks and noting that $a+t$ should be even in order to avoid network families of size zero. Note that $\mathcal{G}^r_{a,b} = \mathbb{S}(G)$ for all $G \in \mathcal{G}^r_{a,b}$, so the choice of input network is moot. We consider only the asymptotic case, where b is large, and denote this family $\mathcal{G}^r_{a,*}$. An example of a network in $\mathcal{G}^3_{9,*}$ is given in Figure S7.

Given a network $G \in \mathcal{G}^r_{a,*}$, let U be the set of sources that have 1 outgoing (non-bidirectional) edge and that V is the set of sources that have r outgoing (non-bidirectional) edges. So $|U| = 2r-2$.

By a counting argument,

$$f_G(M) = A + rB + r^2C,$$

where A is the number of bidirectional edges between two elements in U, B is the number of bidirectional edges between an element in U and an element in V and C is the number of bidirectional edges between two elements in V. This is illustrated in Figure S8.

The number of U-to-sink bidirectional edges is thus $u := |U| - 2A - B$ and the number of V-to-sink bidirectional edges is $v := |V| - 2C - B$. The number of sink-to-sink bidirectional edges is thus $2k := t - u - v$. Hence $k = (t-a)/2 + A + B + C$. These numbers are indicated in Figure S7.

Ignoring single-directional edges, the number of ways of arranging the bidirectional edges to achieve given values of A, B and C is

$$\binom{|U|}{2A, B, u}\binom{|V|}{2C, B, v}\binom{t}{u, v, 2k} \times \frac{(2A)!}{A!2^A}\,\frac{(2C)!}{C!2^C}\,\frac{(2k)!}{k!2^k} \times u!\,v!\,B! = \frac{|U|!\,|V|!\,t!}{A!\,B!\,C!\,2^{A+C+k}\,u!\,v!\,k!},$$

where the computation embodies the idea that nodes in each of U, V and the set of sink nodes have bidirectional edges that must terminate in one of U, V or the set of sink nodes. As such, each of these sets needs to be partitioned into three subsets. This forms the first multiplicand in the computation. The second multiplicand is the number of ways to arrange the bidirectional edges that start and end within the same set (U, V and the set of sinks), and the third multiplicand is the number of ways to arrange the bidirectional edges that cross sets.

Counting the permutations in the previous equation is done by use of Lemma S2.3, which is a well-known result, a proof of which can be found in numerous discrete mathematics texts, e.g. [3, Prop. 3.12].

Lemma S2.3. *The number of permutations of* $\{1, 2, \ldots, n\}$ *with a given cycle structure* π *is given by*

$$C_n(s) = \frac{n!}{\prod_{i\geq 1}\left(i^{s(i)}s(i)!\right)},$$

where $s(i)$ *denotes the number of* i*-cycles in the permutation.*

Ignoring single-directional edges, the total number of ways of arranging the bidirectional edges, which we divide by in order to obtain a probability, is

$$\frac{(a+t)!}{((a+t)/2)!\,2^{(a+t)/2}}.$$

Consider $f_R(M)$ modulo r^2. Knowing $f_R(M)$ modulo r immediately gives the number of bidirectional edges contributing 1 copy of M, because there are exactly $2r-2$ sources that have out-degree 1, so at most $r-1$ bidirectional edges between them. Knowing $f_R(M)$ modulo r^2 gives both the number of bidirectional edges contributing 1 copy of M (as before) and, up to at most two possibilities, the number of bidirectional edges contributing r copies of M. The reason for this is that they must also utilise one of the $2r-2$ sources that have out-degree 1, so their number, known modulo r, is completely determined up to at most two possibilities.

Some values of $f_R(M)$ modulo r^2 are rare, some are impossible altogether, and some may be relatively common. To illustrate:

- To achieve $f_R(M) \equiv -1 \pmod r$, all bidirectional edges with one endpoint in the set U must also have their other endpoint in U. When a is large, the probability of this occurring is small.
- There is no R for which $f_R(M) \not\equiv r-1 \pmod{r^2}$ but $f_R(M) \equiv -1 \pmod r$. The reason for this is that if $f_R(M) \equiv -1 \pmod r$ then all vertices in U have already been used up by bidirectional edges that create 1 copy of M each, and none are left for use by bidirectional edges that create r copies of M each.
- When a is large we can expect $f_R(M)$ values that are equivalent to 0 modulo r to be more common.

This leads to a pattern of peaks and troughs repeating itself every r^2 throughout the distribution of $f_R(M)$. The analytical discussion above shows that events of this type occur repeatedly throughout the tail of $\Pr[f_R(M) = n]$ (See Figure S1).

S2.5 Frequency vs. concentration

In the main paper (in "Frequency vs. concentration") we show that p and p^{conc}, often conflated in the literature, cannot be assumed to be equivalent, or even similar, in the significance orderings they imply. The case considered analytically in the main text is the one claimed in the following theorem.

Theorem S2.4. *Let $G \in \mathcal{F}^{3k}_{k,0,3k}$ be such that each source in G has exactly 3 outgoing edges and each sink has exactly one incoming edge (excluding bidirectional edges). Also, exactly $2m$ of the sinks (but none of the sources) are the endpoint of exactly one bidirectional edge, where the parameter m can be chosen arbitrarily, as long as it is smaller than $\frac{4}{5}k$. For $R \in \mathbb{S}(G)$ and for the induced subgraph $T =$* 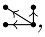*, $c_R(T) \geq c_G(T)$ if and only if $f_R(T) \leq f_G(T)$.*

In this section, we present a proof for this claim.

Proof. The network G is composed of k copies of T, plus m bidirectional edges between the sinks (which are the leaves of the T trees). Each bidirectional edge can either connect two copies of T or two leaves that belong to the same T.

Consider now, for any $R \in \mathbb{S}(G)$, its statistics. Let us say R has a bidirectional edges that are interior to a single copy of T and $m - a$ that connect two copies. Counting $f_R(T)$, we see that this is necessarily $k - a$; of the original k copies, exactly a have been destroyed by bidirectional edges. On the other hand, counting $N_4(R)$, the total number of connected 4-node induced subgraphs in R, we reach $k + 5(m - a)$; in addition to the k copies of T, each inter-copy bidirectional edge adds 5 more components of size 4.

Clearly, $f_R(T)$ decreases monotonously with a. On the other hand,

$$c_R(T) := \frac{f_R(T)}{N_4(R)} = \frac{k-a}{k+5(m-a)}$$

increases monotonously with a. As such, the ordering imposed by $f_R(T)$ for various networks R is exactly the inverse of the order imposed by $c_R(T)$. □

S2.6 Previous criticisms

The introduction of network motifs has received a mostly positive response, although some papers have raised issues with various aspects of the theory and its applications. We summarise the previous criticisms on network motifs as loosely fitting into the following categories:

- Subgraphs in real networks are non-isolated and dynamic [4, 5, 6, 7, 8, 9]; see also [10].
- Frequency does not imply function [11, 12, 13, 14, 15, 16, 17, 18]; see also [19].
- Arguments against theory [20, 21, 22], and implementation [23, 24], particularly overlapping motifs [25, 26, 28]; see also [29, 18, 30].
- Discussions about the effect experimental errors might have on motif detection [31, 32, 27].
- In the case of biological networks, lack of evolutionary pressure for preferential selection of motifs [33, 34, 35, 36]. However, Conant and Wagner [37] consider this an argument for the importance of motifs; see also [38, 39, 40, 41].

There are also numerous methods for modifying the theory of network motifs to deal with its limitations (e.g. [42, 43, 44, 45, 46, 47, 48, 49, 50]). Of this list of criticisms, the following are the most relevant here, since they address theoretical concerns:

- Li, Stones et al. [21] briefly mention that the number of connected digraphs grows extremely fast, and will likely result in insignificant subgraphs being deemed motifs due to multiple hypothesis testing.
- Beber et al. [42] offer a collection of criticisms for the mainstream method for network motif detection, as we do here. For example, they criticise the suitability of the null model which ignores clusters and hierarchy. However, unlike the present work, their approach is largely experimental and directed towards "motif signatures" of metabolic networks.

- Picard et al. [22] have identified that the a normal distribution is, in some cases, a poor approximation (which we also do in Sections S2.1–S2.4). Unlike Picard et al., however, we do not see the replacement of the normal approximation with a compound Poisson approximation as a solution.
- Defoort et al. [27] utilise a heterogeneous gene regulatory network description, where the different molecular interaction types are labelled and create composite network motifs, clustered in biologically relevant modules. They determine that considering the evolutionary age of interactions is more informative for understanding the function of their higher-order topologies than the pure count of these topologies, noting that by comparison standard motif searching proved biased and lacking in multiple dimensions, including high sensitivity to noise and therefore strong dependency on the underlying experimental methodology. They comment that the requirement for degree sequences to remain constant limits the randomisation in network topology. The paper proposes, as an alternative, that, in such a richer network description "The presence of specific network motifs and their aggregation might therefore be a better indicator of biological functionality of a network". However, the paper does not provide any specific alternative criterion that can be applied automatically on massive scales, as is the main purpose of standard motif-finding methods.

S2.7 Anchored motifs

As noted in [27], a major problem in the standard methodology for finding network motifs is that forcing a constant degree sequence creates multiple overlapping constraints that limit the randomisation in the network. We contend that this over-constraining is also the reason behind some of the unintended statistical consequences that we investigate, such as multimodality and inter-motif correlations.

As an alternative, we propose a method based on separating the total motif count into multiple sub-counts, each requiring only a small number of constraints, and then analysing these sub-counts in ways that are not impacted by interactions between the sub-counts.

We define an *anchored motif* to be an induced connected subgraph with one vertex designated as an 'anchor'. One way to separate the count into sub-counts is to partition the appearances of each anchored motif according to the identity of the vertex v of G that is specified as the anchor.

This method allows us to predict many of the phenomena that we see in our study of network motifs. For example, consider the three anchored motifs in Table S2, where the black-coloured vertex indicates the anchor.

$F =$ 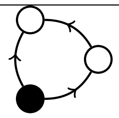	$B =$ 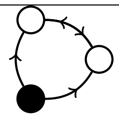	$H =$ 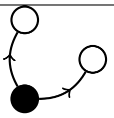
$f_G(F) = 1141$	$f_G(B) = 15$	$f_G(H) = 271,914$

Table S2: Defining the three anchored motifs F, B and H. They are given here together with their motif frequencies in the *E. coli* TF network.

Given any specific vertex, v, if the vertex out-degree is fixed to any number, n, the number of distinct pairs of outgoing edges is $\binom{n}{2}$. For any such pair (v, u) and (v, w), between u and w there may be a one-directional edge, in which case (u, v, w) is an instance of F anchored at v, it may be a bidirectional edge, in which case (u, v, w) is an instance of B anchored at v, or it may be no edge at all, in which case (u, v, w) is an instance of H anchored at v. Enumerating over all such pairs (u, w), we count exactly every instance of F, B and H anchored at v in the *E. coli* TF network. Summing this over all v, we count exactly over all F, B and H. The sum $f_G(F) + f_G(B) + f_G(H)$ depends only on the out-degree sequence of G, and is hence a constant in all networks similar to G. Because the number of bidirectional

edges, $f_G(B)$, in the *E. coli* TF network is small, it is also small in all networks similar to it. As such, the constant total count gives rise to the strong negative correlation between $f_G(F)$ and $f_G(H)$ shown in Figure 5B of the main paper.

This analysis exemplifies a general method for analysing anchored motifs that is not restricted to only F, B and H. To see this, consider as a simple example for a null-hypothesis, what would happen if instead of the standard network motif method of generating networks similar to G, we use the Erdős-Renyi model, which involves alloting every edge independently, using the empirical distribution of G in order to generate each edge.

This, in itself, is not a good null hypothesis, because every motif candidate will show abnormal behaviour in it due to the fact that biological networks tend to display scale free properties [51], which are not exhibited by the Erdős-Renyi model. However, if we count the 3-node motifs anchored at v (for any specific v), only when the edge degrees of v equal their value at G, the behaviour of these counts will be close enough to their actual distribution that in many cases their value at G will not be considered unusual.

For a 3-node motif of a known type, M, anchored at a known v, the number of distinct pairs (u, w) for which (v, u) and (v, w) match M is a known constant, and the remaining (u, w) edges are independent and follow a known distribution. Thus, the statistical behaviour of the number of M anchored at v is completely known: it follows a binomial distribution with known parameters. Correlations between motif counts are also completely known, and in all cases other than those that (like F, B and H) share the description of their edges connecting to v, these correlations are zero.

Thus, if G has an unusual number of occurrences of any M anchored at any v, this can easily be detected through standard statistical testing. The key to this analysis is that when counting the frequency of any M at v, we only constrain the edge degrees of v, not of any other vertex. As such, the counts for distinct vertices are not sampled from the same model (or not sampled from the same set of instances generated from an Erdős-Renyi process). Thus, while the distribution of each sub-count is completely known, the distribution of their sum is not, because the correlations and other interrelations between sub-counts are not known.

However, one can still combine the sub-counts in ways that are not influenced by dependencies. For example, for any statistic that is calculated regarding the frequency of M at v, the expectation of this statistic regarding the frequency of M at a randomly selected vertex is merely the average of the expectations. In particular, the expectation of the count itself can in this way be determined, and we were able to use Markov's inequality in order to determine that three of the 30 possible anchored 3-node motifs are over-represented. Using higher moments in order to detect whether motif counts vary between anchors more than is expected in the null model, we discovered one additional unusual motif.

Another way to combine information between sub-counts, is not to combine them at all but rather to select the one anchor with the best p-value. After ascertaining that 29 of the 30 motifs have an appearance distribution that is the same for each choice of v as an anchor, we considered the best p-value from those choices of v where the potential number of appearances of the motif was largest, so as to get the most representative measurement (noting that because the distribution is known and is binomial, p-values can be computed with confidence and do not rely on implicit normality assumptions). Three of the four previously-identified motifs was re-identified in this way, and an additional 7 unusually frequent motif appearances were found.

These 11 cases all reject the null hypothesis with a p-value of 0.001 or better (and usually tens of orders of magnitude better), and this after rigorous Bonferroni correction to account for multiple hypothesis testing. By contrast, the remaining 19 anchored motifs did not reach even a p-value of 0.05, demonstrating a clear separation between the two sets.

We do not propose the simplistic null hypothesis given here as a direct replacement for network motifs. As a demonstration of its limitations, the 11 high-frequency motifs detected by us can all be traced easily to the degree distribution of the network, specifically to the fact that out-degrees are scale-free distributed

and that bidirectional edges tend to occur at those few vertices that have very high out degrees.

Nevertheless, this example proves that sound statistics can be used to analyse motif frequency, and the 19 motifs that seem to follow the distribution of the null hypothesis demonstrate this fact. For example, the frequencies of anchored motifs F, B and H are all well-predicted by the model, demonstrating no tendency by the network to complete two outgoing edges from a single vertex into a feed-forward motif, and this contrary to the classic results regarding the feed-forward motif in the *E. coli* TF network that started the initial interest in network motifs to begin with.

While our present null model may be simplistic and requires replacement by a more biologically-motivated one, we do believe the use of anchored motifs is a viable alternative to the standard motif analysis methods that offers the potential for rigorous statistical analysis. We leave for follow-up studies the development of better null models for use in future anchored motif research.

S3 Supplementary figures and legends

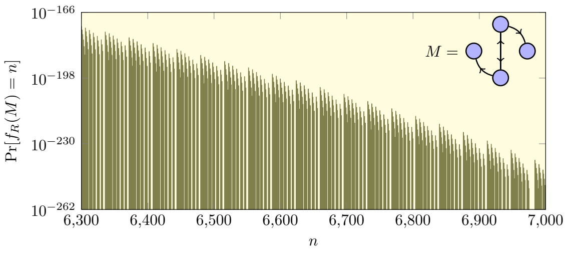

Figure S1: Illustrating analytically the extremely-multimodal behaviour of frequency distributions. The figure shows $\Pr[f_R(M) = n]$ vs. n at two different magnifications; $G \in \mathcal{G}^6_{400,*}$ and $R \in_{\text{uar}} \mathbb{S}(G)$. This behaviour is not captured by the standard p-value, used in mainstream network motif analysis. The data in both graphs are analytically derived and exact, rather than histogram-derived approximations.

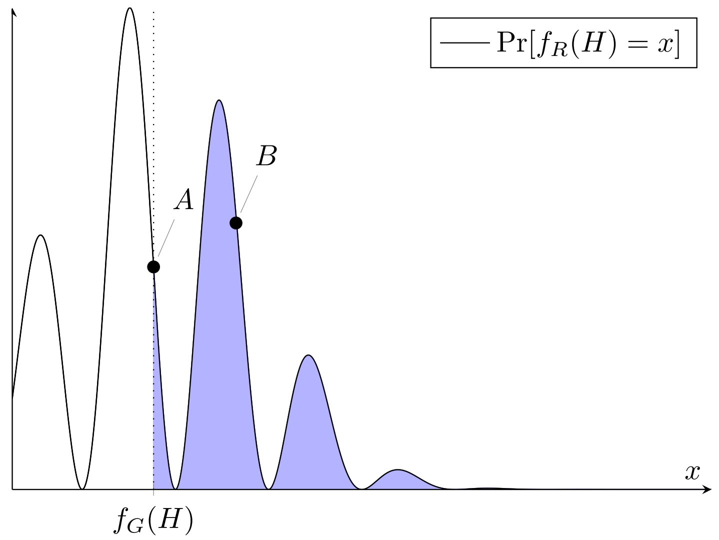

Figure S2: Illustrating a discrepancy that arises because of multimodal distributions. The shaded area indicates the p-value. According to the standard p-value, $f_G(H) = B$ is a more significant phenomenon than $f_G(H) = A$, despite the fact that the former is more likely to be observed randomly.

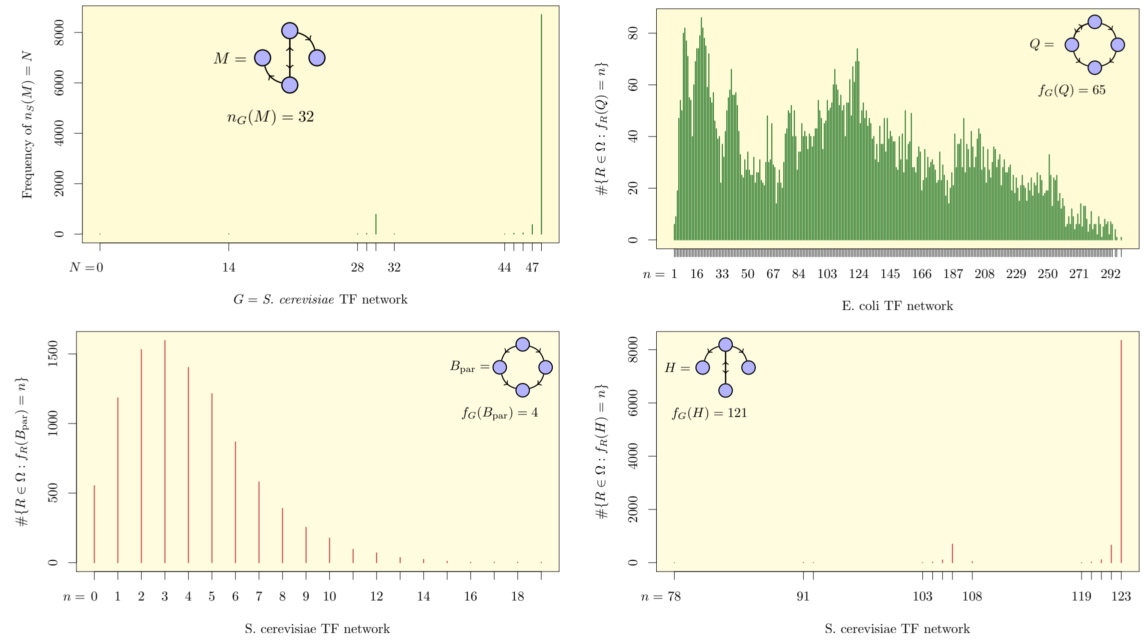

Figure S3: Current best-practices for motif searching implicitly assume that the frequency count, $f_R(H)$, is distributed normally for $R \in_{\text{uar}} \mathbb{S}(G)$. Example histograms of $f_R(H)$ values observed in real-world networks show that this is not a good approximation.

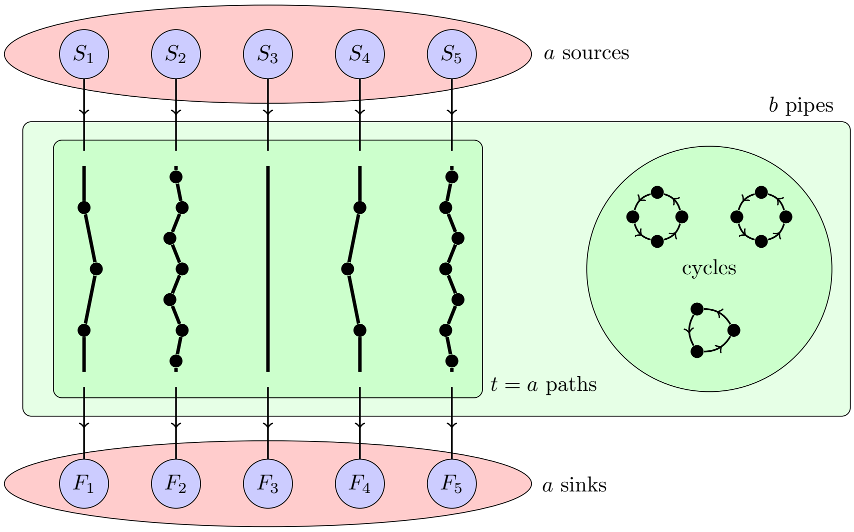

Figure S4: Illustrating how members of $\mathcal{I}_b^t$ give rise to t paths. There are no bidirectional edges in networks in $\mathcal{I}_b^t$.

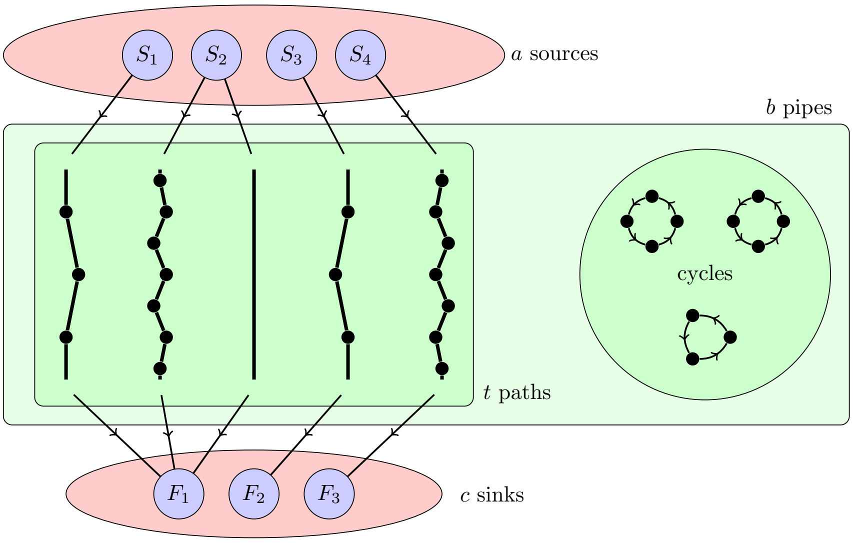

Figure S5: Illustrating how members of $\mathcal{F}^t_{a,b,c}$ give rise to t paths. Bidirectional edges are omitted from the drawing.

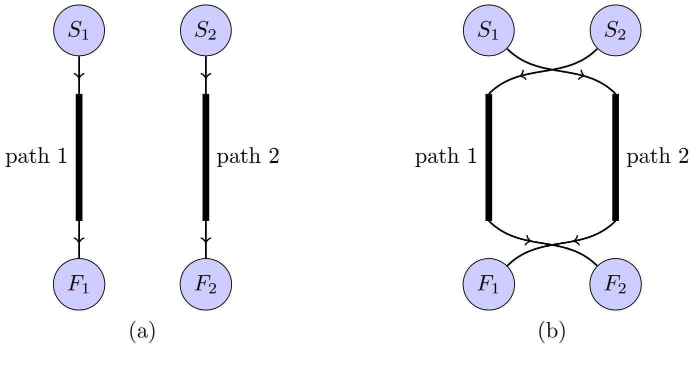

Figure S6: In analysing induced subgraph distributions in members of $\mathcal{F}^t_{a,b,c}$, one must take into account that the t paths are not named. The figure exemplifies how naming the paths allows the same network in $\mathcal{F}^t_{a,b,c}$ to be described in multiple ways.

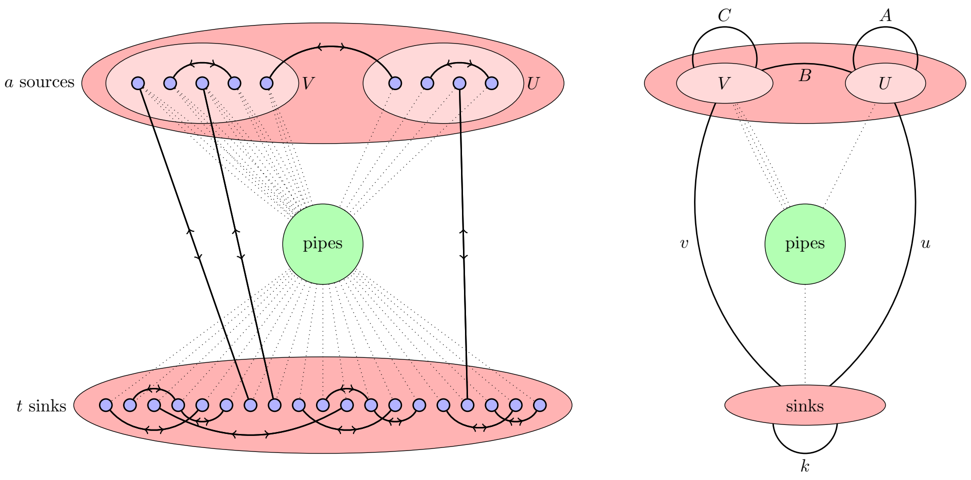

Figure S7: An example of a network in $\mathcal{G}^{3}_{9,*}$ (left) and a diagram illustrating the possible types of bidirectional edges (right). There are 6 possible types of bidirectional edges. The counts of these are denoted by A, B, C, u, v and k.

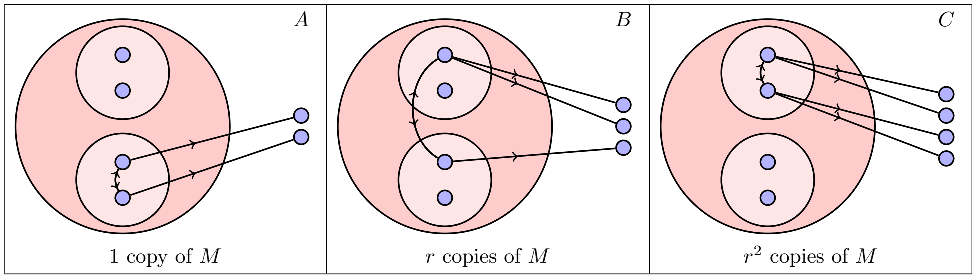

Figure S8: Constructing multimodality analytically: in $\mathcal{G}^{r}_{a,b}$, the configuration of the bidirectional edges determines the number of copies of M modulo r^2.

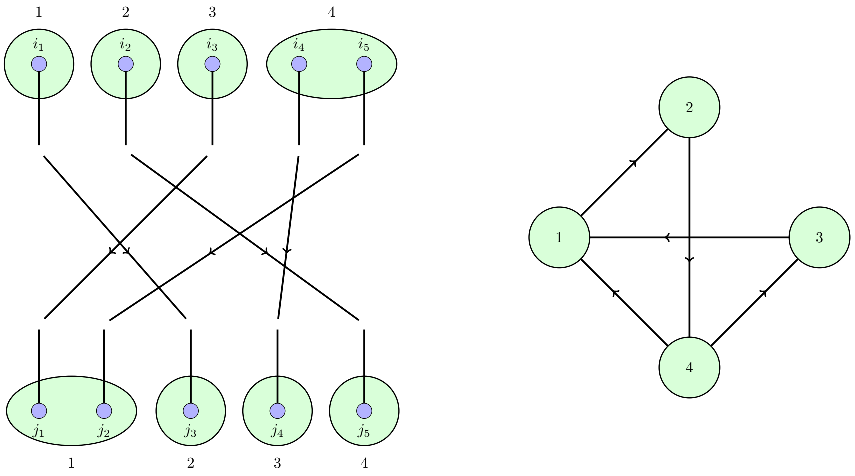

Figure S9: Illustrating the configuration model (see Methods section of manuscript). The graph on the left-hand side is generated from a random permutation. The graph on the right-hand side is generated from the graph on the left by identifying vertices. Unlike the switching method, the configuration model guarantees that all graphs are uniformly and independently sampled. However, this method is usually too slow for practical motif searching.

References

[1] Arratia, R., Barbour, A. D. & Tavaré, S. *Logarithmic combinatorial structures: a probabilistic approach.* EMS Monographs in Mathematics (European Mathematical Society (EMS), Zürich, 2003).

[2] Diaconis, P. & Shahshahani, M. On the eigenvalues of random matrices. *Journal of Applied Probability* **31A**, 49–62 (1994).

[3] Bona, M. *Combinatorics of Permutations* (Chapman and Hall/CRC, 2012), 2 edn.

[4] Hallinan, J. & Jackway, P. T. Network motifs, feedback loops and the dynamics of genetic regulatory networks. In *Proc. IEEE Symposium on Computational Intelligence in Bioinformatics and Computational Biology (CIBCB'05)*, 90–96 (2005).

[5] Hallinan, J. & Wipat, A. Network motifs in context: An exploration of the evolution of oscillatory dynamics in transcriptional networks. In *Proc. IEEE Symposium on Computational Intelligence in Bioinformatics and Computational Biology (CIBCB'08)*, 83–89 (2008).

[6] Harari, O. *et al.* Identifying promoter features of co-regulated genes with similar network motifs. *BMC Bioinformatics* **10** (2009). S1.

[7] Ingram, P. J., Stumpf, M. P. H. & Stark, J. Network motifs: structure does not determine function. *BMC Genomics* **7** (2006).

[8] Knabe, J. F., Nehaniv, C. L. & Schilstra, M. J. Do motifs reflect evolved function?—No convergent evolution of genetic regulatory network subgraph topologies. *Biosystems* **94**, 68–74 (2008).

[9] Macía, J., Widder, S. & Solé, R. Specialized or flexible feed-forward loop motifs: a question of topology. *BMC Bioinformatics* **3** (2009).

[10] Prill, R. J., Iglesias, P. A. & Levchenko, A. Dynamic properties of network motifs contribute to biological network organization. *PLoS Biology* **3** (2005). E343.

[11] Apte, A. A., Cain, J. W., Bonchev, D. G. & Fong, S. S. Cellular automata simulation of topological effects on the dynamics of feed-forward motifs. *J. Biol Eng.* **2** (2008).

[12] Artzy-Randrup, Y., Fleishman, S. J., Ben-Tal, N. & Stone, L. Comment on "Network motifs: Simple building blocks of complex networks" and "Superfamilies of evolved and designed networks". *Science* **305**, 1107 (2004).

[13] Goemann, B., Wingender, E. & Potapov, A. P. An approach to evaluate the topological significance of motifs and other patterns in regulatory networks. *BMC Systems Biology* **3** (2009).

[14] Konagurthu, A. S. & Lesk, A. M. On the origin of distribution patterns of motifs in biological networks. *BMC Systems Biology* **2** (2008).

[15] Mazurie, A., Bottani, S. & Vergassola, M. An evolutionary and functional assessment of regulatory network motifs. *Genome Biology* **6** (2005).

[16] Meshi, O., Shlomi, T. & Ruppin, E. Evolutionary conservation and over-representation of functionally enriched network patterns in the yeast regulatory network. *BMC Systems Biology* **1** (2007).

[17] Taylor, D. T. *et al.* Toward a classification of isodynamic feed-forward motifs. *J Biol. Dyn.* **4**, 196–211 (2010).

[18] Vázquez, A. *et al.* The topological relationship between the large-scale attributes and local interaction patterns of complex networks. *Proc. Natl. Acad. Sci. USA* **101**, 17940–17945 (2004).

[19] Milo, R., Itzkovitz, S., Kashtan, N., Levitt, R. & Alon, U. Response to comment on "Network motifs: Simple building blocks of complex networks" and "Superfamilies of evolved and designed networks". *Science* **305**, 1107 (2004).

[20] Konagurthu, A. S. & Lesk, A. M. Single and multiple input modules in regulatory networks. *Proteins* **73**, 320–324 (2008).

[21] Li, X. *et al.* NetMODE: Network motif detection without Nauty. *PLoS ONE* **7** (2012).

[22] Picard, F., Daudin, J. J., Koskas, M., Schbath, S. & Robin, S. Assessing the exceptionality of network motifs. *J. Comput. Biol.* **15**, 1–20 (2008).

[23] Artzy-Randrup, Y. & Stone, L. Generating uniformly distributed random networks. *Phys. Rev. E* **72**, 056708 (2005).

[24] Ginoza, R. & Mugler, A. Network motifs come in sets: Correlations in the randomization process. *Phys. Rev. E* 011921 (2010).

[25] Cheng, C.-Y., Huang, C.-Y. & Sun, C.-T. Mining bridge and brick motifs from complex biological networks for functionally and statistically significant discovery. *IEEE Trans. Syst. Man Cybern. B Cybern.* **38**, 17–24 (2008).

[26] Schreiber, F. & Schwöbermeyer, H. Towards motif detection in networks: frequency concepts and flexible search. In *Proc. International Workshop on Network Tools and Applications in Biology (NETTAB'04)*, 91–102 (2004).

[27] Defoort, J. & Van de Peer, Y. & Vermeirssen, V. Function, dynamics and evolution of network motif modules in integrated gene regulatory networks of worm and plant. *Nucleic acids research* **46**, 6480–6503 (2018).

[28] Grochow, J. & Kellis, M. Network motif discovery using subgraph enumeration and symmetry-breaking. In *Proc. 11th conference on Research in Computational Molecular Biology (RECOMB'07)*, 92–106 (2007).

[29] Dobrin, R., Beg, Q. K., Barabási, A.-L. & Oltvai, Z. N. Aggregation of topological motifs in the Escherichia coli transcriptional regulatory network. *BMC Bioinformatics* **5** (2004).

[30] Zhang, L. V. *et al.* Motifs, themes and thematic maps of an integrated Saccharomyces cerevisiae interaction network. *J. Biol.* **4** (2005).

[31] Ma, H.-W. *et al.* An extended transcriptional regulatory network of Escherichia coli and analysis of its hierarchical structure and network motifs. *Nucleic Acids Res.* **32**, 6643–6649 (2004).

[32] Marbach, D. *et al.* Revealing strengths and weaknesses of methods for gene network inference. *Proc. Natl. Acad. Sci. USA* **107**, 6286–6291 (2010).

[33] Dwight Kuo, P., Banzhaf, W. & Leier, A. Network topology and the evolution of dynamics in an artificial genetic regulatory network model created by whole genome duplication and divergence. *Biosystems* **85**, 177–200 (2006).

[34] Solé, R. V. & Valverde, S. Are network motifs the spandrels of cellular complexity? *Trends Ecol. Evol.* **21**, 419–422 (2006).

[35] Solé, R. V. & Valverde, S. Spontaneous emergence of modularity in cellular networks. *J. R. Soc. Interface* **5**, 129–133 (2008).

[36] Valverde, S. & Solé, R. V. Network motifs in computational graphs: A case study in software architecture. *Phys. Rev. E* **72** (2005). 026107.

[37] Conant, G. C. & Wagner, A. Convergent evolution of gene circuits. *Nat. Genet.* **34**, 264–266 (2003).

[38] de Silva, E. & Stumpf, M. P. H. Complex networks and simple models in biology. *J. R. Soc. Interface* **2**, 419–430 (2005).

[39] Kim, J.-R., Yoon, Y. & Cho, K.-H. Coupled feedback loops form dynamic motifs of cellular networks. *Biophysical Journal* **94**, 359–365 (2008).

[40] Lagomarsino, M. C., Bassetti, B., Castellani, G. & Remondini, D. Functional models for large-scale gene regulation networks: realism and fiction. *Mol. Biosyst.* **5**, 335–344 (2009).

[41] Wuchty, S., Oltvai, Z. N. & Barabási, A.-L. Evolutionary conservation of motif constituents in the yeast protein interaction network. *Nature Genetics* **35**, 176–179 (2003).

[42] Beber, M. E. *et al.* Artefacts in statistical analyses of network motifs: general framework and application to metabolic networks. *J. R. Soc. Interface* **7**, 3426–3435 (2012).

[43] Berg, J. & Lässig, M. Local graph alignment and motif search in biological networks. *Proc. Natl. Acad. Sci. USA* **101**, 14689–14694 (2004).

[44] Chechik, G. *et al.* Activity motifs reveal principles of timing in transcriptional control of the yeast metabolic network. *Nat Biotechnol.* **26**, 1251–1259 (2008).

[45] Geard, N., Bullock, S., Lohaus, R., Azevedo, R. B. R. & Wiles, J. Developmental motifs reveal complex structure in cell lineages. *Complexity* **16**, 48–57 (2011).

[46] Jiang, R., Tu, Z., Chen, T. & Sun, F. Network motif identification in stochastic networks. *Proc. Natl. Acad. Sci. USA* **103**, 9404–9409 (2006).

[47] Jin, R., McCallen, S. & Almaas, E. Trend motif: A graph mining approach for analysis of dynamic complex networks. In *Proc. 7th IEEE International Conference on Data Mining*, 541–546 (2007).

[48] Kim, W., Li, M., Wang, J. & Pan, Y. Biological network motif detection and evaluation. *BMC Bioinformatics* **5** (2011). S5.

[49] Onnela, J.-P., Saramäki, J., Kertész, J. & Kaski, K. Intensity and coherence of motifs in weighted complex networks. *Phys. Rev. E* **71** (2005). 065103(R).

[50] Saramäk, J., Onnela, J.-P., Kertész, J. & Kaski, K. Characterizing motifs in weighted complex networks. *AIP Conference Proceedings* **776**, 108–117 (2005).

[51] Barabási, Albert-László and Albert, Réka *Emergence of scaling in random networks Science* **286**, 509–512 (1999).
